# MicroRNA Profiling during Craniofacial Development: Potential Roles for *Mir23b* and *Mir133b*

**DOI:** 10.3389/fphys.2016.00281

**Published:** 2016-07-14

**Authors:** Hai-Lei Ding, Joan E. Hooper, Peter Batzel, B. Frank Eames, John H. Postlethwait, Kristin B. Artinger, David E. Clouthier

**Affiliations:** ^1^Department of Craniofacial Biology, School of Dental Medicine, University of Colorado Anschutz Medical CampusAurora, CO, USA; ^2^Department of Cell and Developmental Biology, School of Medicine, University of Colorado Anschutz Medical CampusAurora, CO, USA; ^3^Department of Neuroscience, University of OregonEugene, OR, USA; ^4^Department of Anatomy and Cell Biology, University of SaskatchewanSaskatoon, SK, Canada

**Keywords:** craniofacial development, neural crest cell, mouse, zebrafish, RNA duplex, facebase

## Abstract

Defects in mid-facial development, including cleft lip/palate, account for a large number of human birth defects annually. In many cases, aberrant gene expression results in either a reduction in the number of neural crest cells (NCCs) that reach the frontonasal region and form much of the facial skeleton or subsequent failure of NCC patterning and differentiation into bone and cartilage. While loss of gene expression is often associated with developmental defects, aberrant upregulation of expression can also be detrimental. microRNAs (miRNAs) are a class of non-coding RNAs that normally repress gene expression by binding to recognition sequences located in the 3′ UTR of target mRNAs. miRNAs play important roles in many developmental systems, including midfacial development. Here, we take advantage of high throughput RNA sequencing (RNA-seq) from different tissues of the developing mouse midface to interrogate the miRs that are expressed in the midface and select a subset for further expression analysis. Among those examined, we focused on four that showed the highest expression level in *in situ* hybridization analysis. *Mir23b* and *Mir24.1* are specifically expressed in the developing mouse frontonasal region, in addition to areas in the perichondrium, tongue musculature and cranial ganglia. *Mir23b* is also expressed in the palatal shelves and in anterior epithelium of the palate. In contrast, *Mir133b* and *Mir128.2* are mainly expressed in head and trunk musculature. Expression analysis of *mir23b* and *mir133b* in zebrafish suggests that *mir23b* is expressed in the pharyngeal arch, otic vesicle, and trunk muscle while *mir133b* is similarly expressed in head and trunk muscle. Functional analysis by overexpression of *mir23b* in zebrafish leads to broadening of the ethmoid plate and aberrant cartilage structures in the viscerocranium, while overexpression of *mir133b* causes a reduction in ethmoid plate size and a significant midfacial cleft. These data illustrate that *miRs* are expressed in the developing midface and that *Mir23b and Mir133b* may have roles in this developmental process.

## Introduction

Morphogenesis of the vertebrate face is a complex event requiring coordination among a variety of signaling cascades. Human craniofacial birth defects occur at a world-wide rate of 1:800 births (Schutte and Murray, 1999; Spritz, 2001), with structural defects often resulting from failure of spatio-temporal integration of these signaling cascades. While the vertebrate mid-face has a complex embryonic origin, most facial birth defects result from disruption of cranial neural crest cell (NCC) patterning and differentiation (Chai and Maxson, 2006; Knight and Schilling, 2006; Walker and Trainor, 2006; Dixon et al., 2011; Clouthier et al., 2013). Cranial NCCs arise at the border between the neural and non-neural ectoderm and subsequently migrate to the frontonasal region (Le Douarin, 1982; Bronner-Fraser, 1995). These cells eventually give rise to most of the bone, cartilage, and connective tissue of the mid-face and neck (Couly et al., 1996; Köntges and Lumsden, 1996). NCC development relies on intricate regulation of patterning cues within specific boundaries of the head. While boundaries are established through continuous refinement of gene expression, the mechanisms required for this refinement are less clear.

MicroRNAs (miRNAs) are a class of small, noncoding RNAs that inhibit translation of target mRNAs by binding to a recognition sequence almost always in the 3′ untranslated region (UTR) of the target mRNA (Lee et al., 1993; Wightman et al., 1993; Lai, 2002; Lewis et al., 2003). This binding results in decreased mRNA translation through a number of mechanisms that can include cleavage and degradation of the target mRNA, translational repression, deadenylation of the 3′-poly(A) tail and thus mRNA decay and miRNA sponging (Hutvágner et al., 2001; Zheng and Cullen, 2003; Wu et al., 2006; Hausser and Zavolan, 2014; Jens and Rajewsky, 2015). miRNAs have been implicated in many clinically relevant processes, including development, cancer, and stem cell maintenance and differentiation (Chen et al., 2012; Fernández-Hernando et al., 2013; Kuppusamy et al., 2013; Morceau et al., 2013; Parpart and Wang, 2013). miRNAs are also involved in numerous aspects of craniofacial development, including palatogenesis, odontogenesis, and upper and lower jaw development (Tavares et al., 2015). One of the first examples of miRNA action in facial development is *mir140*, whose regulation of *pdgfra* is required for NCCs to migrate past the optic stalk on their way from the hindbrain to the future palate (Eberhart et al., 2008). A SNP in the human *MIR140* gene leads to reduced *Mir140* expression in murine palatal mesenchymal cells (Li et al., 2011). In addition, this SNP is associated with increased risk of nonsyndromic cleft palate in mothers who smoke during pregnancy (Li et al., 2010). These findings suggest that miRNA function is evolutionarily conserved and illustrates a role for miRNAs in human palate development. Another miRNA family involved in craniofacial development is the *MIR17* and *MIR92* family, which has been linked to Feingold syndrome in human patients (Kannu et al., 2013; Tassano et al., 2013). Targeted knockouts of *Mir17 and Mir92* in mice results in hypoplasia of most skull bones, including reduced ossification and cleft palate, similar to human patients (Ventura et al., 2008; de Pontual et al., 2011; Li et al., 2012; Wang et al., 2013). In addition, others, such as *Mir196, Mir199*, and *Mir20*0 have likely roles in determining craniofacial size, bone and cartilage formation, and cartilage size, respectively (Watanabe et al., 2008; Desvignes et al., 2014). However, targeted deletion of the miRNA processing enzyme DICER in NCCs results in hypoplasia of most craniofacial structures, suggesting that numerous other miRNAs are also likely involved in this process (Huang et al., 2010; Zehir et al., 2010; Nie et al., 2011; Oommen et al., 2012).

Here, we use data from a high-throughput miRNA sequencing project of developing mouse facial structures to identify many miRNAs that are potentially involved in craniofacial development. We have examined the expression of a number of these miRNAs in both mouse and zebrafish. Further, we have performed functional analysis of four of these miRNAs in zebrafish. Our *in situ* hybridization and overexpression analyses provide evidence that *Mir23b* and *Mir133b* are important regulators of craniofacial development.

## Materials and methods

### miRNA-Seq differential expression analysis

RNA-Seq count data with gene assignments have been deposited in the FaceBase repository for craniofacial research (www.facebase.org). Specific FaceBase accession numbers are: E10.5 FB00000662.01, E11.5 FB00000663.01, E12.5 FB00000664.01, FB00000664.01, E13.5 FB00000665.01, E14.5 FB00000666.01. Counts of overlapping and nested sequences assigned to each mature (1035 sequences) or hairpin (306 sequences) mouse miRNA were summed to obtain consolidated counts per mature or hairpin miRNA (724 and 255, respectively). A threshold of 1 was used for the RNA-seq analysis. After low filtering (mean counts per sample >5; 629 mature miRs and 163 hairpin miRNAs), we used DESeq to identify miRNAs differentially expressed by anatomy or age. For principal components analysis and hierarchical clustering, count data was normalized using the regularized log transformation in the DESeq R package. A *p* < 0.01 after multiple testing correction (Benjamini and Hochberg method) was used, as well as no fold-change threshold (Benjamini and Hochberg, 1995).

### Mouse and zebrafish maintenance

All mouse embryo collection was performed using 129S6 mice (Taconic), with the day of the copulation plug denoted as 0.5 days. Embryo collection and fixation were performed as previously described (Clouthier et al., 1998). Zebrafish were maintained according to common lab practice (Westerfield, 2000), with embryo staging according to established methods (Kimmel et al., 1995). The wild type lines used are AB and an in-cross line maintained between the AB and TL lines (TAB). This study was carried out in accordance with the recommendations of the Institutional Animal Care and Use Committee (IACUC) of the University of Colorado Anschutz Medical Campus as laid out in protocols approved by IACUC. Nomenclature for miRNAs is consistent with that described in Desvignes et al. (2015). For simplicity, pri-MiR(mouse) and pri-miR (zebrafish) were annotated without the “pri” in the text below.

### Cloning and probe synthesis

To detect expression of mouse MiRNAs, we used both Locked Nucleic Acid (LNA; Obernosterer et al., 2007) and conventional riboprobes against the primary *pri-MiR* transcripts, both labeled with digoxigenin (He et al., 2011). For LNA-based experiments, Exiqon LNA *Mir133b* (#616614-360) and *Mir23b* (#615366-360) probes were used. To generate *pri-MiR* probes, PCR primers were designed to amplify about 200–600 nt of the primary MiR transcript centered on the mature miRNA sequence for mouse and zebrafish. All primer sequences are given in Supplemental Table 1. To generate mouse *pri-MiRs*, PCR primers were used with genomic tail DNA obtained from tails of 2 month old mice. To generate zebrafish *pri-miRs*, PCR primers were used with 5 day post fertilization (dpf) zebrafish embryos. PCR products were cloned into the pCR®II-TOPO® Vector (Invitrogen). Inserts were validated by sequencing. Plasmids were linearized and transcribed using the DIG Labeling Kit (Roche). Probes were purified as previously described (Clouthier et al., 1998). The opposite stand sense probes were used as controls for specificity for the antisense expression (data not shown).

### Mouse and zebrafish whole mount *in situ* hybridization (ISH)

Whole mount ISH in mouse was performed as previously described (Clouthier et al., 1998). For sectional miRNA ISH, serial 10 μm frontal sections through the head of E12.5 and/or E13.5 mouse embryos were collected on Superfrost Plus slides (Fisher). Subsequent ISH was performed as previously described (Hendershot et al., 2007). Hybridizations were performed at 70⋅C overnight. Colorimetric detection was performed with BM purple (Roche). Signal detection required an average of 2–3 days in staining solution. After developing, slides were rinsed in TBST, coverslipped and photographed using Nomarski optics on an Olympus BX51 compound microscope fitted with a DP71 digital camera. Whole-mount ISH in zebrafish was performed as previously described (Johnson et al., 2011). BM Purple (Roche) was used as a substrate for the alkaline phosphatase reaction. After staining, embryos were mounted in 80% glycerol or 3% methylcellulose and imaged under Nomarski optics on the Olympus BX51 compound microscope as described for mice.

### Overexpression analysis and bone and cartilage staining

MiRNA duplexes were created by annealing 5p and 3p MiRs together at 95⋅C and slowly cooling to room temperature (MiR133b-3p: UUUGGUCCCCUUCAACCA; MiR133b-5p: GCUGGUCAAAUGGAACCA; MiR23b-3p: AUCACAUUGCCAGGGAUU; MiR23b-5p: GGUUCUUGGCAUGCUGA). Thirty-three micrometers of MiR23b duplex (5′—3′), 6.25 μM of MiR133b (5′—3′), and 33 μM of standard control miRNA (5′- CTTACCTCAGTTACAATTTATA -3 duplexed with 5′- TAAATTGTAACTGAGGTAAGAG-3′) were injected into single cell zebrafish embryos and allowed to grow for 6 dpf. Zebrafish embryos were fixed and stained with alizarin red and alcian blue (Birkholz et al., 2009) with minor modifications. Larvae were fixed in 2% PFA/PBS for 1 h at room temperature, washed in 100 mM Tris (pH 7.5)/10 mM MgCl_2_, and stained overnight in 0.04% alcian blue (Anatech Ltd) in 80% EtOH/100 mM Tris (pH 7.5)/10 mM MgCl_2_. Larvae were then rehydrated in 80% EtOH/100 mM Tris (pH 7.5)/10 mM MgCl_2_, followed by 50 and 25% EtOH/100 mM Tris (pH 7.5), cleared in 25% glycerol/0.1% KOH, then stained 30 min with 0.01% alizarin red (Sigma) in 25% glycerol/100 mM Tris (pH 7.5) and stored in 50% glycerol/0.1% KOH. Stained skeletons were dissected, flat-mounted and imaged as previously described (Johnson et al., 2011).

## Results

### Identification of miRNAs involved in midfacial development from miRNA-Seq data

**Figure 1:**
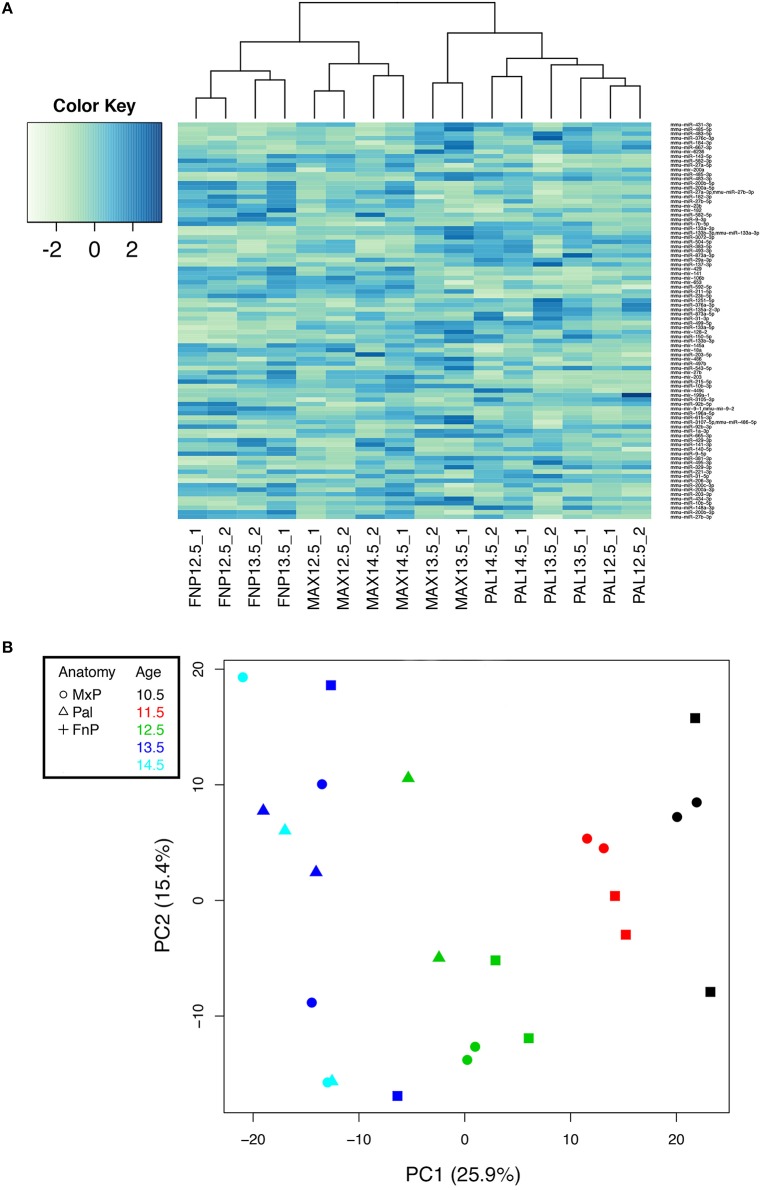
**Analysis of miRNA-Seq data defines miRNAs whose expression differs by prominence. (A)** 49 mature miRNAs and 11 hairpin miRNAs were differentially expressed by prominence (FDR adjusted *p* < 0.01). Hierarchical clustering of their normalized and scaled expression values (using the R hclust function, ward. D2 method) shows groups of miRNAs enriched in the frontonasal prominence (FnP), palate (Pal), the maxillary prominence (Max), or in combinations thereof. The dendrogram at the top shows the clustering of the samples; the three major divisions correspond to FnP, Pal, and Max, and duplicate samples cluster together. **(B)** Principal Component Analysis (PCA) analysis of data. Data is color coded by age (inset), with tissue source denoted by symbol (inset). Principal Component 1 (PC1) distributes samples by age. Principal Component 2 (PC2) is orthogonal to PC1 but does not correspond to any obvious property of the samples.

**Figure 2:**
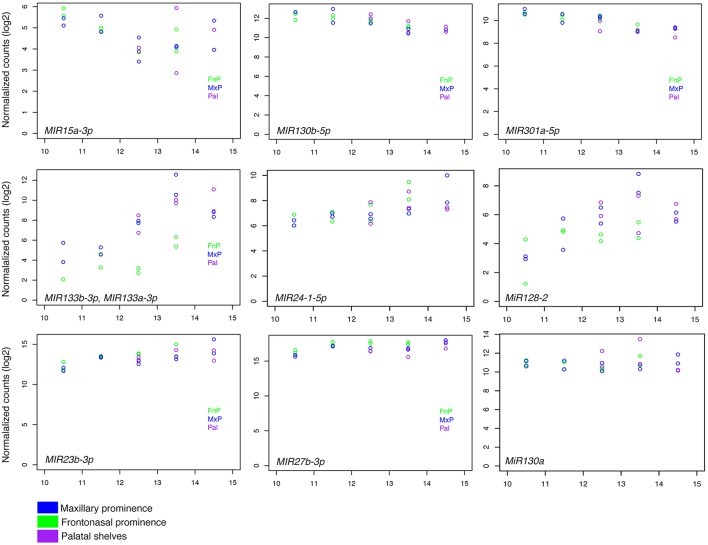
**RNAseq values for expression of selected miRNAs**. Normalized counts for selected miRNAs from each prominence (see inset) as compared to embryonic age (E10–15). Each row represents a different pattern of expression over time. **(Top)** *Mir15a, Mir130b*, and *Mir301a* showed a downward trend of expression over time. **(Middle)** Expression of *Mir133b, Mir24.1*, and *Mir1282* increased over time. **(Bottom)** *Mir23b, Mir27b*, and *Mir130a* were expressed consistently over time. Blue, Maxillary prominence; Green, Frontonasal prominence; and Purple, Palate.

We have previously conducted massively parallel miRNA sequencing (miRNA-Seq) on miRNAs extracted from embryonic age (E) 10.5, E11.5, E12.5, E13.5, and E14.5 mouse maxillary prominences, frontonasal prominences, and palatal shelves, with the data deposited in FaceBase (www.facebase.org). Between E10.5 and E11.5, NCC-derived mesenchyme that will give rise to craniofacial structures undergoes significant patterning events that establishes the positional and functional identity of the mesenchyme. Between E12.5 and E14.5, gene expression in the facial region is driving differentiation into the bone and cartilage of the upper and lower face. Because we are interested in the mechanisms that govern this second event, we focused our analysis of miRNA expression between E12.5 and E14.5. Using a combination of biostatical approaches and packages (Section Materials and Methods), we identified 262 differentially expressed mature miRNAs and 71 hairpin miRNAs at a false discovery rate-adjusted *p*>0.01 (data not shown). Of these, 49 mature miRNAs and 11 hairpin miRNAs were differentially expressed between maxillary prominences, frontonasal prominences, and palatal shelves (Figure 1A); some also differed by age (Figure 2). Table 1 shows the top differentially expressed miRNAs as total counts, while Figure 2 shows the log2fold change in expression of specific miRNAs between facial prominences at E13.5. Total counts of each miRNA are also shown in Table 1 so that the relative expression of miRNAs can be compared.

**Table 1:** **microRNAs that are differentially expressed between prominences at E13.5**.

	**Normalized counts**						
**microRNA**	**Fn (1)**	**Fn (2)**	**Mx (1)**	**Mx (2)**	**Pal (1)**	**Pal (2)**	**Pattern**
*MIR1a-3p*	911.78	711.95	**9059.94**	**11523.71**	**3472.3**	**4385.36**	Pal&Mx
*MiR128.2*	45.21	20.06	**444.7**	**181.49**	**157.72**	**24.72**	Pal&Mx
*MIR133a-3p*	67.82	165.42	**3063.12**	**1421.33**	**628.5**	**722.18**	Pal&Mx
*MIR133a-5p*	11.3	23.03	**225.67**	**249.28**	**47.79**	**186.84**	Pal&Mx
*MIRp133b-3p*	0	10.9	**560.85**	**203.36**	**109.93**	**186.37**	Pal&Mx
*MIR133b-3p, MIR133a-3p*	41.44	77.51	**5883.99**	**1478.18**	**1049.1**	**781.84**	pAL&Mx
*MIR184-3p*	97.96	97.57	**829.67**	**467.95**	**389.53**	**285.26**	Pal&Mx
*MIR206-3p*	678.18	281.07	**26993.99**	**23655.32**	**13105.35**	**10618**	Pal&Mx
*MIR214-3p*	14901.2	6127.98	**19507.09**	**17443.01**	**20518.33**	**28066.97**	Pal&Mx
*MIR483-3p*	331.56	253.33	**1158.21**	**682.24**	**924.83**	**584.54**	Pal&Mx
*MIR499-5p*	15.07	25.01	**235.62**	**308.32**	**52.57**	**156.18**	Pal&Mx
*MIR3099-3p*	90.42	48.54	**132.75**	**124.64**	**136.22**	**154.51**	Pal&Mx
*MIR137-3p*	56.52	67.36	**185.84**	**67.79**	**143.38**	**911.87**	Pal>Mx
*MIR483-5p*	350.39	151.8	**487.84**	**756.59**	**642.84**	**1184.77**	Pal>Mx
*let-7e-5p*	3142.25	3950.77	2216.87	5053.38	**4698.23**	**15721.97**	Pal
*MIR31-3p*	11.3	37.64	19.91	21.87	**64.52**	**221.07**	Pal
*MIR31-5p*	877.87	1350.11	365.05	531.36	**1464.91**	**4273.87**	Pal
*MIR135a-2-3p*	18.84	22.29	46.46	54.67	**114.71**	**150.47**	Pal
*MIR376c-3p*	184.62	181.02	348.46	419.84	**291.55**	**841.27**	Pal
*MIR450a-2-3p*	3.77	29.22	6.64	4.37	**23.9**	**68.46**	Pal
*MIR495-3p*	565.15	1077.21	1181.44	1176.42	**996.52**	**2931.26**	Pal
*MIR500-3p*	470.96	430.88	418.15	750.03	**630.89**	**1213.3**	Pal
*MIR873a-3p*	94.19	61.91	146.02	67.79	**611.77**	**54.44**	Pal
*MIR873a-5p*	60.28	75.78	23.23	41.55	**157.72**	**174.24**	Pal
*MIR1251-5p*	26.37	91.38	89.6	61.23	**133.83**	**434.07**	Pal
*MIR7b-5p*	**768.61**	**226.09**	49.78	61.23	**1032.37**	**305.46**	Fn>Pal
*MIR200a-3p*	**13639.02**	**20365.01**	2598.51	3391.51	**3345.64**	**13267.57**	Fn&Pal
*MIR9-3p*	**968.3**	**1467.98**	89.6	104.96	71.69	139.54	Fn
*MIR9-5p*	**7490.16**	**4400.23**	726.79	763.15	365.63	487.32	Fn
*MiR23b*	**380.54**	**244.91**	79.65	104.96	86.03	71.31	Fn
*MiR141*	**259.97**	**162.7**	26.55	48.11	26.29	51.35	Fn
*MiR200a*	**862.8**	**569.81**	149.34	150.88	303.5	66.32	Fn
*MIR200b-3p*	**141254.31**	**59360.85**	7055.47	17631.06	15461.64	23046.91	Fn
*MIR200b-5p*	**779.91**	**289.98**	73.01	96.21	164.89	87.48	Fn
*MIR3085-3p*	**3.77**	**29.72**	13.27	8.75	7.17	9.75	Fn
*MIR429-3p*	**5218.24**	**10519.04**	1085.2	1445.38	1930.91	3339.65	Fn
*MiR653*	**195.92**	**80.98**	33.19	102.77	69.3	63.71	Fn
*Mir19b.1*	**184.62**	**198.11**	**59.74**	**170.56**	50.18	146.67	Fn> Mx
*MiR27b*	**64.05**	**23.77**	**19.91**	**8.75**	4.78	5.94	Fn> Mx
*MIR200a-5p*	**678.18**	**249.12**	**79.65**	**87.47**	95.59	62.99	Fn> Mx
*MiR429*	**327.79**	**131.99**	**39.82**	**76.53**	28.68	43.03	Fn> Mx
*MIR215-5p*	**75.35**	**54.97**	**6.64**	**13.12**	4.78	3.09	Fn> Mx
*MiR18a*	**33.91**	**36.9**	**26.55**	**15.31**	14.34	28.29	Fn & Mx
*MIR18b-5p*	**52.75**	**48.29**	**13.27**	**32.8**	9.56	64.9	Fn & Mx
*MIR20b-5p*	**437.05**	**646.33**	**172.57**	**321.44**	114.71	422.89	Fn & Mx
*MIR27b-5p*	**440.82**	**129.02**	**73.01**	**181.49**	64.52	78.68	Fn & Mx
*MiR92a.2*	**832.66**	**1542.27**	**1025.47**	**848.43**	1493.59	657.99	Fn & Mx
*MIR106a-5p*	**290.11**	**252.84**	**69.69**	**177.12**	33.46	192.07	Fn & Mx
*MiR106b*	**128.1**	**70.82**	**49.78**	**59.04**	23.9	22.82	Fn & Mx
*MIR182-3p*	**395.61**	**350.16**	**126.11**	**65.6**	148.16	99.36	Fn & Mx
*MIR293-3p*	**11.3**	**11.39**	**13.27**	**13.12**	4.78	10.7	Fn & Mx
*MIR302a-5p*	**30.14**	**20.06**	**26.55**	**34.99**	11.95	23.77	Fn & Mx
*MIR302b-3p*	**18.84**	**85.68**	**53.1**	**39.36**	33.46	70.84	Fn & Mx
*MIR302d-3p*	**48.98**	**84.2**	**46.46**	**72.16**	43.02	117.67	Fn & Mx
*MiR326*	**26.37**	**26.5**	**29.87**	**26.24**	23.9	7.84	Fn & Mx
*MiR3473f*	**7.54**	**9.66**	**13.27**	**26.24**	0	5.71	Fn & Mx
*MIR582-3p*	**391.84**	**241.69**	**574.13**	**301.76**	210.3	69.89	Fn & Mx
*MiR6399*	**26.37**	**4.46**	**9.96**	**4.37**	2.39	1.19	Fn & Mx
*MiR6539*	**45.21**	**13.12**	**16.59**	**30.61**	14.34	15.93	Fn & Mx
*MIR18b-3p*	**0**	**4.21**	**0**	**0**	2.39	0.95	Mx>Fn
*MIR128.1-5p*	**90.42**	**33.93**	**172.57**	**48.11**	64.52	28.76	Mx>Fn
*MIR211-5p*	**131.87**	**119.11**	**242.26**	**74.35**	21.51	15.93	Mx>Fn
*MIR216b-5p*	**26.37**	**52.99**	**33.19**	**8.75**	2.39	19.97	Mx>Fn
*MIR217-5p*	**22.61**	**21.3**	**13.27**	**30.61**	2.39	7.84	Mx>Fn
*MIR363-3p*	**252.43**	**832.8**	**504.44**	**284.27**	446.88	173.53	Mx>Fn
*MiR486*	**26.37**	**6.44**	**132.75**	**83.09**	28.68	6.89	Mx>Fn
*MIR741-3p*	**3.77**	**2.72**	**23.23**	**0**	0	1.66	Mx>Fn

*Colored and bolded areas indicate groups of miRs that are highly expressed within a specific prominence. Green, Frontal nasal prominence (Fn); Brown, Maxilla (Mx); Pink, Palate (Pal)*.

### Principal components analysis of miRNAs expressed in the face

Principal Components Analysis (PCA) is a primary quality control assay, with the expectation that replicates will cluster and that principal components might reflect known qualities of the sample conditions (e.g., age, anatomy) or of the experiment (e.g., batch or read depth). After sequence consolidation and low-expression filtering, normalized counts for 979 miRNAs (724 mature miRNAs and 255 hairpin miRNAs) were used to analyze the principal components of the variability among the 24 samples (Figure 1B). The first principal component, which accounts for 26% of the variability, distributed the samples by age (color-coded). Neither prominence (symbols) nor read depth (not shown) correlated with any of the top five principal components. Biological duplicates from E10.5-E12.5 tended to cluster, indicating biological reproducibility at those ages. Together these demonstrate the biological reproducibility for key miRNAs between replicates and within prominences.

### Expression analysis of selected miRNAs in the mouse at E12.5

For our *in situ* hybridization (ISH) analysis of miRNA expression in the developing midface, we focused on E12.5 mouse embryos, as at this stage, the lateral and medial nasal prominences, palatal shelves, and maxilla are well-defined and are undergoing extensive growth. We initially examined miRNA expression in E12.5 mouse embryo using whole mount ISH and LNA probes against *Mir23b, Mir24.1*, and *Mir666* (Supplemental Figure 1). LNA probes specifically detect the mature Mir sequence. However, the overall level of expression of these Mir's as detected in whole mount analysis was low (likely due in part to poor probe penetration; Supplemental Figure 1), leading us to examine Mir expression using frozen sectional ISH analysis. When using LNA probes against *Mir23b* and *Mir133b*, robust expression was present in a variety of facial structures, though overall background staining on the sections was high (Supplemental Figure 2). To next assess whether we could improve the signal to noise ratio of the staining, we PCR-generated probes against the pri-miRNA transcript, an approach we have previously used to examine Mir expression in embryos (He et al., 2011). Probes encompassed the pri-miRNA sequence (see (Section) Materials and Methods). These probes detect both pri-miRNAs and mature miRNAs, so expression is expected in both the nucleus and cytoplasm. However, the relative abundance of mature miRNAs for any single species compared to that of pri-miRNA species makes it far more likely that these probes detect the mature miRNA. Further, we have shown that the use of pri-miRNA probes for miRNA ISH provides comparable results to those using LNA probes (He et al., 2011). Based on our bioinformatics analysis (Figures 1, 2), we examined the expression of 13 pri-miRNAs in E12.5 mouse embryos and found that many were expressed in specific embryonic tissues, including facial structures (Table 2 and Supplemental Figures 3–9). Based on this initial analysis of ISH data, we focused our subsequent analysis on *MiR23b* and *MiR133b*.

**Table 2:** **Expression profiles of miRNAs in the developing mouse midface**.

**miRNA**	**Embryonic age**	**Expression profile**
*Mir15a*	E12.5	Tongue, oral and nasal epithelium, nasal mesenchyme
*Mir20a*	E12.5	Eye, lung, brain
*Mir23b*	E12.5, E13.5	Naris, vibrissae, TG, DRG, palatal shelf, maxilla, tongue, nasal and tongue epithelium, tongue muscle, incisor, otic capsule, malleus, diencephalon, limb muscle, mesothelium, intervertebral space, foregut, gastrointestinal neurons
*Mir24.1*	E12.5, E13.5	Vibrissae, TG, DRG, nasal epithelium, maxilla, tongue, incisor, limb muscle, gastrointestinal neurons, mesothelium, intervertebral space, pinna
*Mir27b*	E12.5	Tongue, limb, rib, pinna, TG, nasal epithelium, eye, vibrissae, brain, facial cartilage, intestinal neuron
*Mir128.2*	E12.5, E13.5	Maxilla, TG, DRG, tongue, diencephalon, telencephalon, ocular muscles, limb, mesothelium, ribs
*Mir130a*	E12.5	Tongue, rib, limb, intestine, facial cartilage, nasal epithelium, maxilla
*Mir130b*	E12.5	Tongue, intestine, facial cartilage, nasal epithelium, maxilla
*Mir133b*	E12.5, E13.5	Vibrissae, maxilla, TG, tongue, intervertebral space, hyoid, ocular muscles, diencephalon, muscle, mesothelium, ribs
*Mir206*	E12.5	Tongue, rib, limb, eye, maxilla, nasal epithelium
*Mir335*	E12.5	Tongue, brain
*Mir376*	E12.5	Tongue, rib, mandible, vertebrae, lateral nasal prominence
*Mir411*	E13.5	Tongue, proximal mandible and mesenchyme of the outflow tract, maxillary mesenchyme
*Mir666*	E12.5, E13.5	FNP, DRG, ocular muscle, tongue, mandible, vertebrae, mesothelium, ribs, liver, spinal cord, limb

### Expression of *MiR23b* and *MiR133b* in mouse facial structures at E12.5

**Figure 3:**
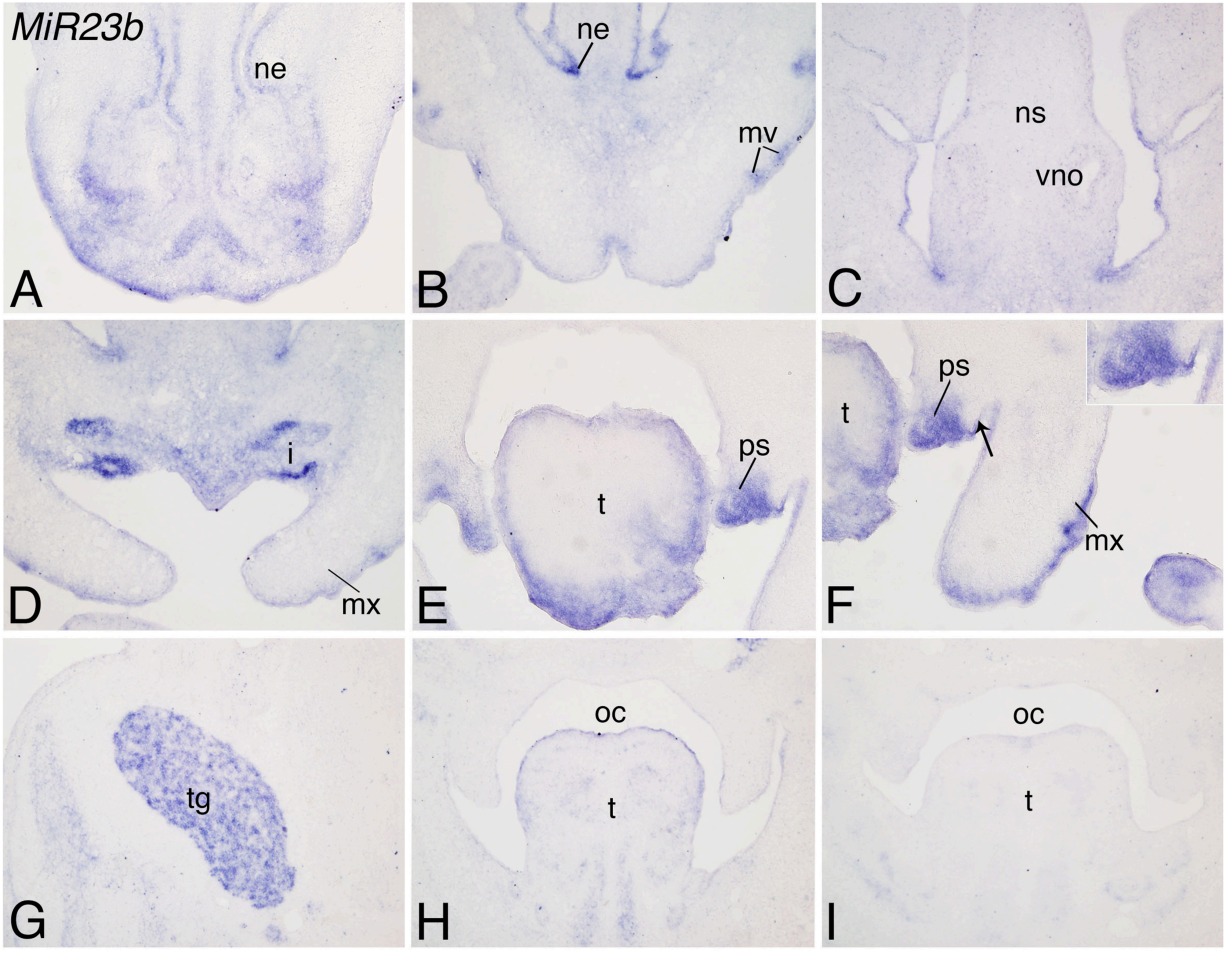
**Expression of *Mir23b* in mouse facial structures at E12.5**. ISH analysis in frozen frontal sections through the head of E12.5 mouse embryos. **(A–C)** *Mir23b* is moderately expressed in the nasal epithelium epithelium (ne), mystacial vibrissae (mv), nasal septum (ns), and vomeronasal organ (vo). **(D–F)** *Mir23b* is also expressed in the upper incisors (i), tongue (t), maxillary epithelium (te), and palatal shelves (ps). The arrow in **(F)** denotes the epithelium where *Mir23b* expression begins. **(G–I)** More posteriorly, expression is observed in the trigeminal ganglion (tg; **G**), posterior tongue (t; **H,I**). i, incisor; mx, maxilla; oc, oral cavity.

In E12.5 wild type mouse embryos, *MiR23b* expression appeared throughout the facial region including the nasal epithelium, mystacial vibrissae, and vomeronasal nasal organs (Figures 3A–C), upper incisors (Figure 3D), tongue connective tissue and epithelium (Figures 3E,F), and maxillary epithelium (Figure 3F). Expression was also observed in the anterior palatal shelves, with expression more prominent on the nasal side of the shelves (Figures 3E,F) and in the trigeminal ganglion (Figure 3G). In addition, *MiR23b* was expressed along the palatal shelf epithelium, again starting at the midline of the shelf and continuing on the oral side (Figures 3H,I). Overall, the expression pattern of *MiR23b* supported our analysis (Figure 1), though it was difficult to assess the qualitative differences in expression between prominences compared to the quantitative differences of miRNA-seq. In the genome, *MiR23b* is part of the 850 bp *Mirc23* cluster, which contains *Mir23b, Mir27b*, and *Mir3074.1*. In addition, *Mir24.1* is 5 kb away from *Mir23b*. As previously described for many clustered miRNAs (Lagos-Quintana et al., 2003; Lim et al., 2003), *Mir24.1* had an expression pattern similar to that observed for *Mir23b*, including expression in the nasal epithelium (Supplemental Figures 3A–C), tongue (Supplemental Figures 3E,F,H,I) and maxillary process epithelium (Supplemental Figure 3D), though expression in the palatal shelf mesenchyme and overlying epithelium (Supplemental Figures 3D,F,H,I) and trigeminal ganglia (Supplemental Figure 3G) was weak.

**Figure 4:**
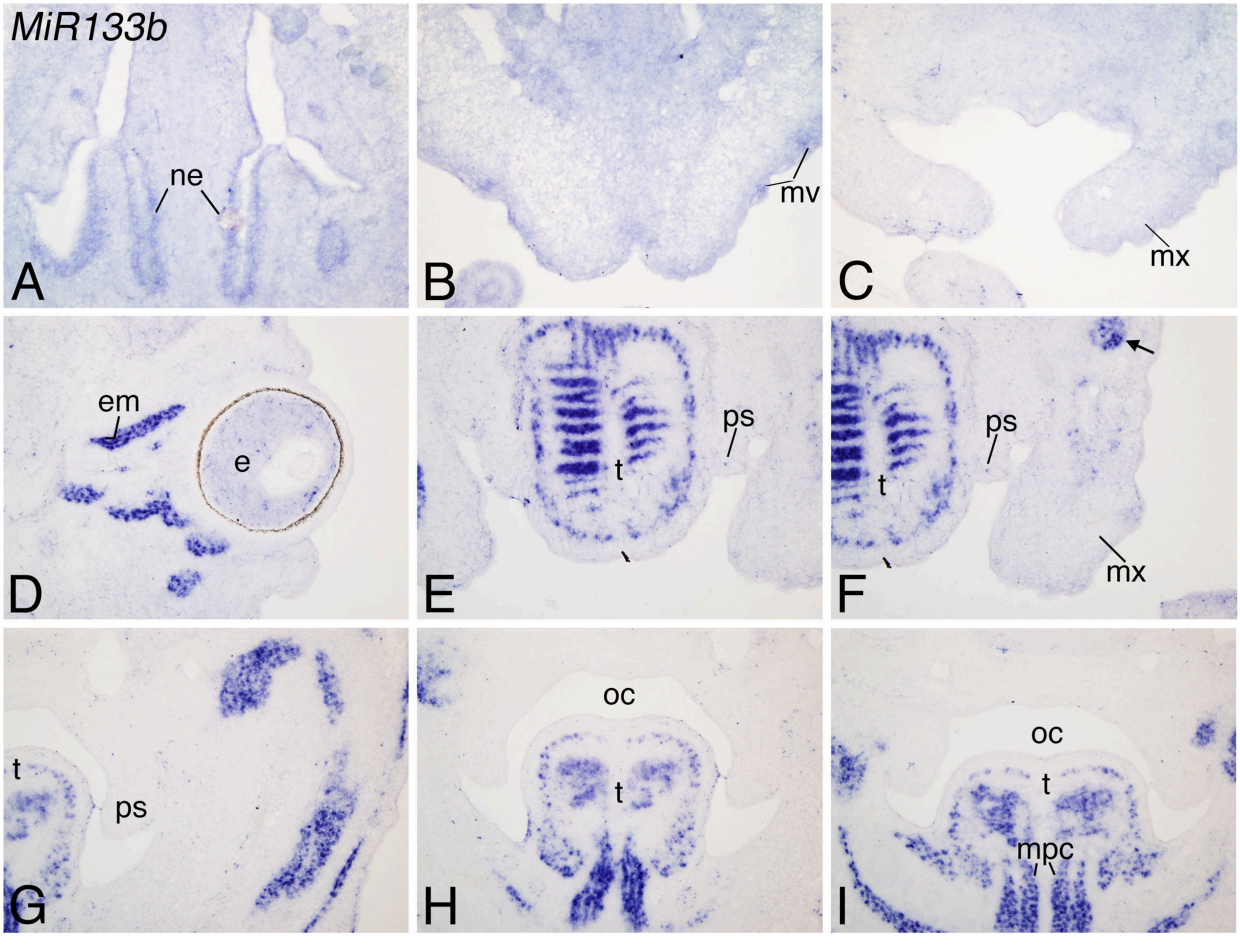
**Expression of *Mir133b* in mouse facial muscles at E12.5**. ISH analysis in frozen frontal sections through the head of E12.5 mouse embryos. **(A–C)** *Mir133b* is weakly expressed in the nasal epithelium (ne; **A**), mystacial vibrissae (mv; **B**), and maxilla (mx); **(D–F)**. (mx); **(D–F)**. *Mir133b* is strongly expressed in facial muscles, including the masseter (ma), and in the muscles of the eye (em; **D**) and tongue muscle (t; **E–I**) Expression is not observed in the palatal shelves (ps; **E,F**). **(G–I)** Posterior expression continues continues in the tongue muscles. e, eye; oc, oral cavity.

Like *MiR23b, MiR133b* was also strongly expressed in the craniofacial region at E12.5. Weak expression was observed in the nasal epithelium, mystacial vibrissae and maxilla (Figures 4A–C), but strong expression was observed in facial musculature, including the intrinsic and extrinsic musculature of the tongue and eye (Figures 4D–I) and facial muscles (including the masseter muscle (arrow; Figure 4F). Expression of *MiR133b* was not observed in the maxilla or palatal shelves, suggesting that the expression observed by miRNA-seq might reflect presence of other tissue in dissected samples. Like *Mir23b, Mir133b* exists in a cluster with *Mir206*, which had a similar pattern of expression to that of *Mir133b*. Message was primarily detected in facial muscles (Supplemental Figures 4E,F), with message also detected in the cornea and lens of the eye (Supplemental Figure 4A). Further, like the comparison between *MiR23b* and *MiR24.1*, expression of *MiR206* was much weaker than the expression observed for *MiR133b*.

### Expression of *mir23b* and *mir133b* is conserved in zebrafish facial structures

**Figure 5:**
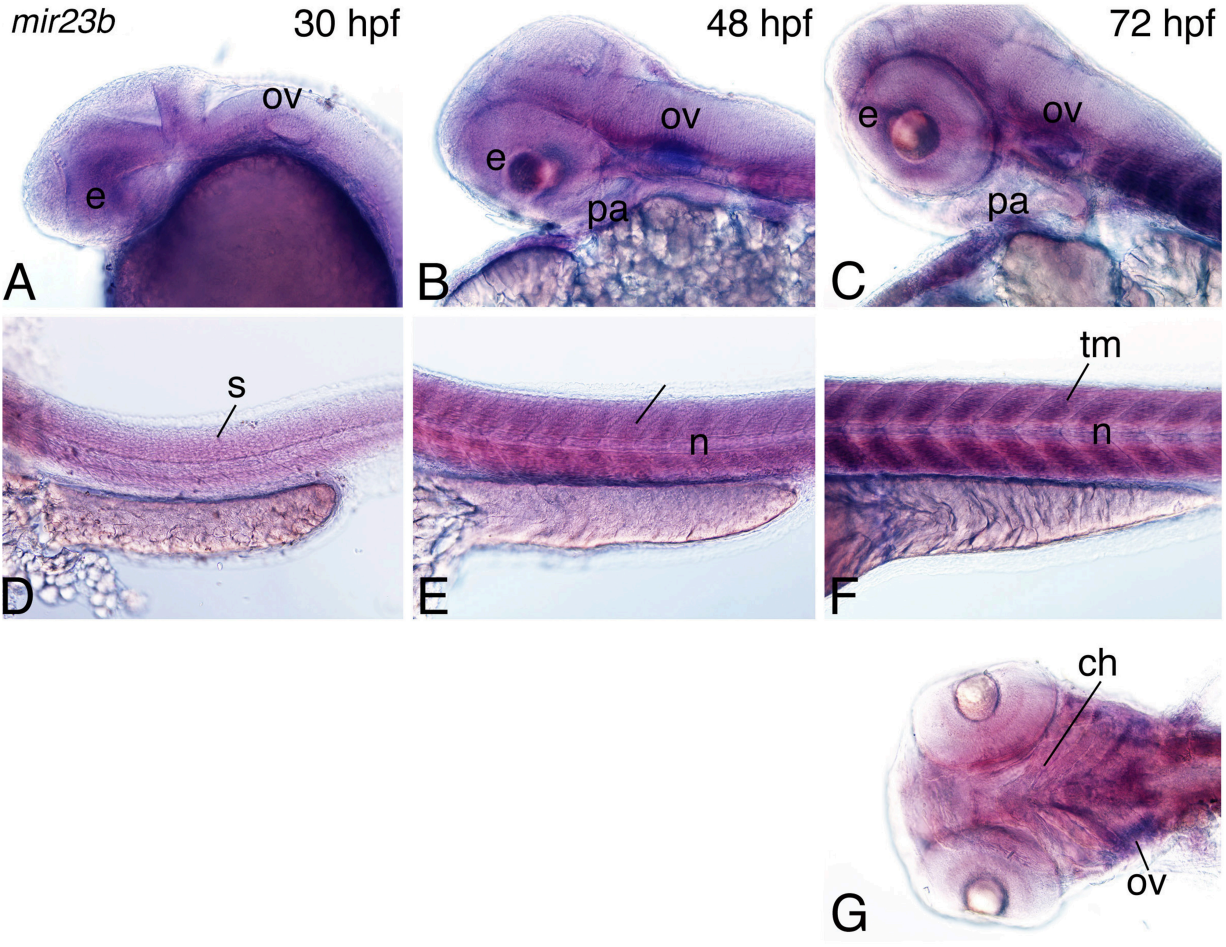
**Expression of mir23b in zebrafish embryos**. Whole-mount *in situ* hybridization analysis with a digoxigenin-labeled probe against the *mir23b* transcript at 30–72 hpf. **(A–C)** Expression is observed in the otic vesicle (ov; **A**), weakly in the eye (e), and pharyngeal arch **(B,C)**, and palate (pl; **C**). **(D–F)** In the trunk, expression is observed in the trunk muscle the somites (s; **E,D**) at 30, 48 hpf, and trunk muscle (tm; **F**) at 72 hpf. **(G)** Ventral view of expression shows more detailed expression in the cranial muscle including the muscles supporting the craniofacial structures (arrows) and the eye. Anterior is to the left. n, notochord; ch, ceratohyal.

Based on analysis of *mir140* action, miRNA function during facial development is also present in zebrafish embryos. Similarly, we found that a selected subset of the miRNAs from Table 1 were also expressed in the head region of 30-hours post fertilization (hpf) zebrafish embryos (Table 3 and Supplemental Figure 10). To examine whether the pattern of expression of *mir23b* and *mir133b* was also conserved between mouse and zebrafish embryos, we examined expression of both miRNAs in 30–72 hpf embryos. Expression of *mir23b* was detected in the head and pharyngeal arch mesenchyme (Figure 5A) and in the somitic mesoderm (Figure 5D) at 30 hpf. Diffuse expression was also present in the eye (Figure 5A). At 48 hpf, *mir23b* expression was still present in the head and pharyngeal arch mesenchyme while also appearing in the otic vesicle (Figure 5B). Expression was also observed in the trunk muscle and notochord (Figure 5E). By 72 hpf, expression remained in cranial muscle (Figures 5C,G) while also being present in the somite-derived trunk muscle and notochord (Figure 5F) and in ceratobranchial structures (arrows, Figure 5G).

**Table 3:** **Expression profiles of miRNAs in the developing zebrafish**.

**miRNA**	**Embryonic age**	**Expression profile**
*mir15a*	48 and 72 hpf	Midbrain, MHB, notochord
*mir15b*	48 and 72 hpf	Midbrain, neurocranium, notochord
*mir23b*	30, 48, and 72 hpf	Somites, lens, pharyngeal arches, notochord
*mir27b*	48 and 72 hpf	
*mir30c*	48 and 72 hpf	Brain, neurocranium, eye, heart
*mir130a*	48 and 72 hpf	Brain, gut tube, heart, eye
*mir133b*	30, 48, and 72 hpf	Notochord
*mir301a*	48 and 72 hpf	Forming cartilage
		Midbrain, neurocranium, eye, trigeminal ganglia

**Figure 6:**
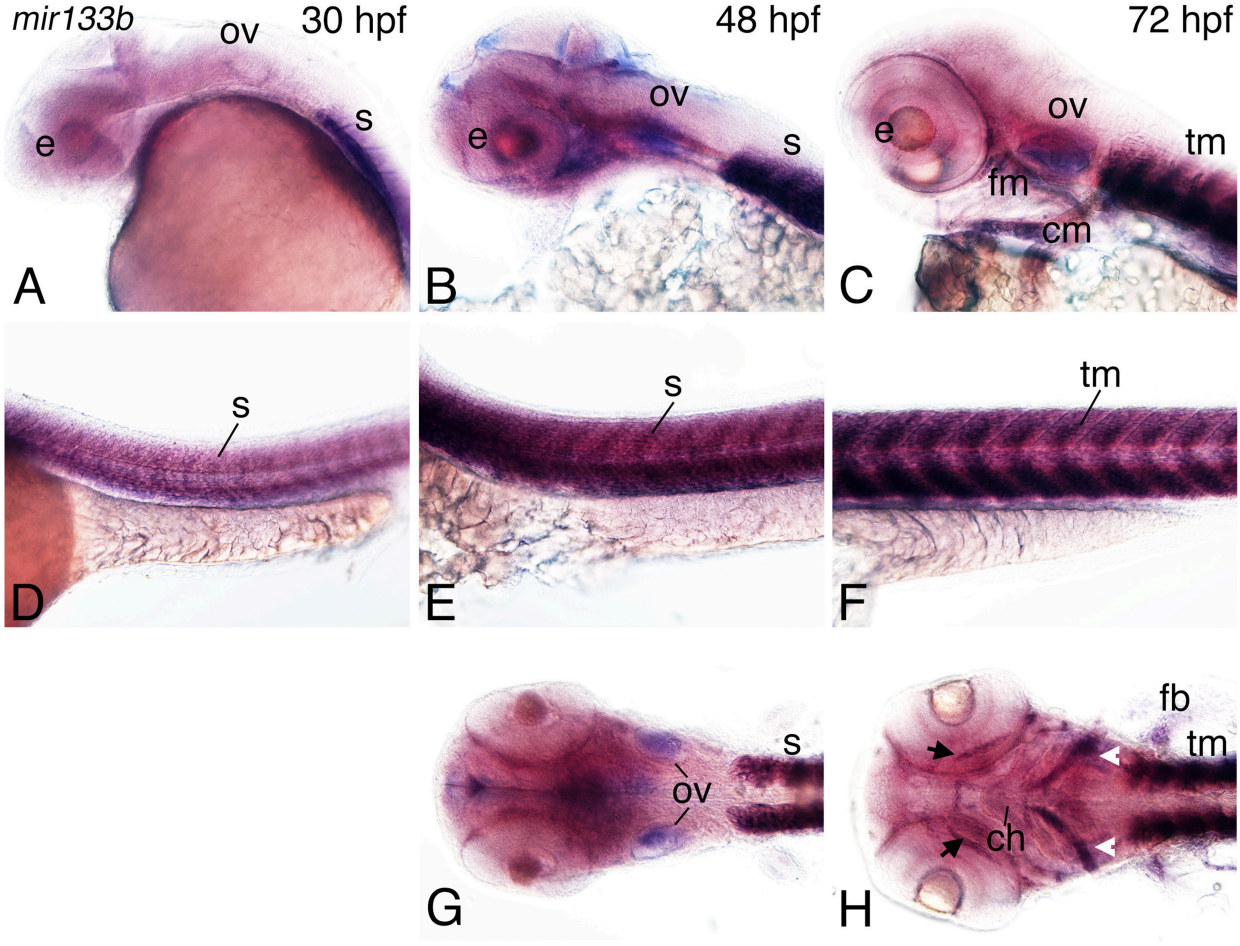
**Expression of *mir133b* in zebrafish embryos**. Whole-mount *in situ* hybridization analysis with a digoxigenin-labeled probe against *mir133b* at 30–72 hpf. **(A–C)** Expression is observed in the developing pharyngeal region, including at 48 hpf in the facial muscle (fm) and otic vessicle **(B,G)**. **(D–F)** Strong expression of *miR133b* is observed in the somites (s; **D**) at 30 hpf, somites **(E)**, and pectoral fin (pf) at 48 hpf **(H)** and facial muscle (fm; **C**), cardiac muscle (cm; **C**), and trunk muscle (tm; **F**) at 72 hpf, including the anterior mandibularis (arrows) and stemohyoideus (arrowheads) facial muscles muscles. Anterior is to the left. e, eye; tm, trunk muscle; n, notochord; ch, ceratohyal.

At 30 hpf, *mir133b* expression was also observed in the head region (around the eye and portions of the brain; Figure 6A), though expression was weaker than that of *mir23b*. Expression was quite strong in the developing somites (Figure 6D). By 48 hpf, expression around the otic vesicle, eye, and posterior brain was increased (Figures 6B,G). As in 30 hpf embryos, *mir133b* was strongly expressed in the somites (Figure 6E). By 72 hpf, *mir133b* expression was observed in trunk muscles (Figure 6F), otic vesicle and heart (Figure 6C). Expression was also present in the facial muscles, including the anterior mandibularis (arrow, Figure 6H) and muscles developing near the ceratobranchials, including the stemohyoideus (arrowheads, Figure 6H).

### *mir23b* and *mir133b* overexpression results in viscerocranial and neurocranial defects in zebrafish

**Figure 7:**
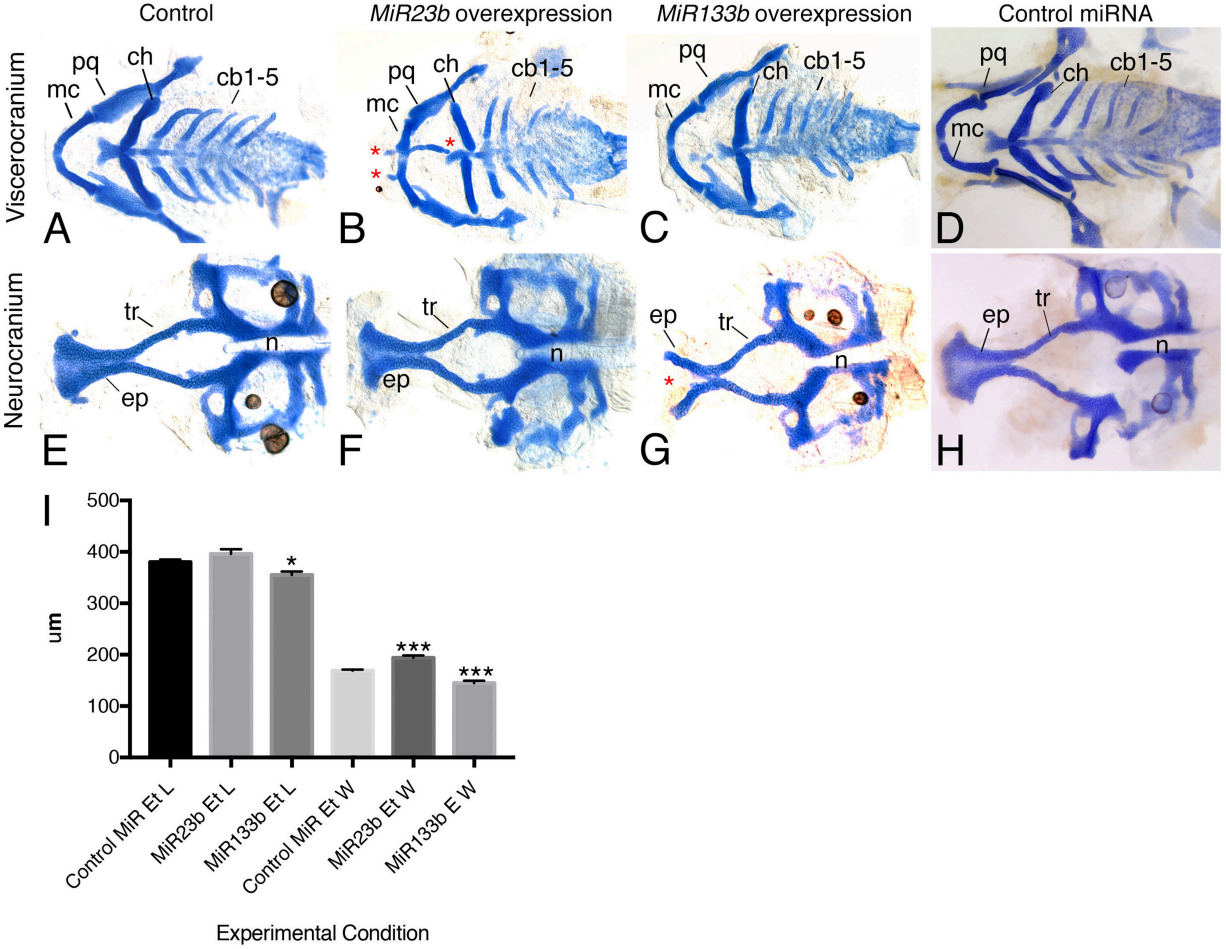
**Injection of MiR133b and MiR23b RNA duplexes resulted in facial defects**. Alcian blue stain of 6 dpf zebrafish larvae, flat mounted ventral views, anterior is to the left. **(A,D,E,H)** Control embryos, either uninjected or 33 μM control miR-injected, display wild type type craniofacial cartilage in both the viscerocranium and neurocranium. **(B,F)** Injection of 33 μM RNA duplex of MiR23b leds to a slight increase in the width of the ethmoid plate, likely resulting from shortening of the trabeculae (tr) **(F)**, and production of ectopic cartilage (^*^; **B**) in the **B**) in the viscerocranium. **(C,G)** Injection of 6.25 μM RNA duplex of MiR133b resulted in hypoplastic cartilage structure and a cleft (^*^) in the ethmoid plate (ep; **G,I**)**. (I)** Quantification of ethmoid plate length and width comparing standard control MiR (*n* = 10) injected with 33 μM compared to MiR23b (*n* = 9) and MiR133b (*n* = 9). Elements were measured and the mean and standard deviation for each are: Ethmoid plate length for control MiR, 380 ± 13.23 μm; MiR23b, 396 ± 27.13 μm; and MiR133b, 355 ± 19.27 μm; Ethmoid plate width for control MiR, 169.2 ± 4.92 μm; MiR23b, 193.9 ± 193.9 ± 14.09 μm; and MiR133b*:* 144.7 ± 14.7μm. Student's (Welsh's) *T*-test was used to compare to the standard control using GraphPad PRISM. ^*^p < 0.02 and ^***^p < 0.0008. Anterior is to the left. cb, ceratobranchial; ch, ceratohyal; mc, Meckel's cartilage; n, notochord; pq, palatoquadrate.

Two potential methods for assessing function of genes in zebrafish are over-expression and gene inactivation. While Crispr-Cas9-mediated gene inactivation is underway, we began our analysis of potential function by injecting 1–2 cell zebrafish embryos with duplex RNA for MiR23b and MiR133b examining cartilage development at 6 dpf. Overexpression of miRNA duplex is more likely to generate a phenotype, as increasing the repressor function will result in reduced gene expression of the miRNA gene targets. In the viscerocranium, MiR23b duplex injection resulted in aberrant development of Meckel's cartilage and the ceratohyal (Figure 7B). In addition, ectopic cartilage extended from the basihyal to the medial aspect of Meckel's cartilage (23/128; 18%; Figure 7B). Two bilateral ectopic cartilages also extended anteriorly from Meckel's cartilage. Morphological changes were also present in the neurocranium, including a slight increase in the width of the ethmoid plate likely resulting from a shortening of the trabeculae (74/128; 58%; Figures 7F,I) as compared to standard control MiR injected embryos (*n* = 10; Figures 7D,H,I) and control non-injected embryos (Figures 7A,E).

Injection of duplex RNA for MiR133b resulted in the formation of a cleft in the ethmoid plate (40/89; 45%; Figure 7G) and a general reduction in the overall size of the neurocranium, with a significant reduction in both the ethmoid plate width and length (46/89; 52%; Figures 7G,I). Injected animals also showed a mild change in the viscerocranium, including a reduction in the size of the ceratohyal (Figure 7C).

## Discussion

miRNAs have well defined roles in numerous developmental process, including craniofacial development (Tavares et al., 2015). We have shown here that numerous miRNAs are expressed within the developing midface, with expression patterns for some of these suggesting distinct functions in a variety of tissues. In addition, we have shown that over-expression of *mir23b* and *mir133b* results in changes in craniofacial cartilage morphogenesis. Together, results from our study illustrate the utility of an approach to quickly assess potential miRNA function during vertebrate morphogenesis.

### Identification of miRNAs in craniofacial development

In addition to the identification of novel miRNAs expressed in the craniofacial complex, several miRNAs identified in this study as differentially expressed between facial prominences and over time have been identified to play a role in development in other systems. *Mir199a*, which we found to be enriched in the palate as compared to the FNP at E13.5, is involved in aspects of chondrogenesis and osteogenesis (Suomi et al., 2008; Lin et al., 2009). Similarly, *Mir92a* which is in the *Mirc1* cluster on mouse chromosome 14 containing *Mir17, Mir18, Mir19a, Mir20a, Mir19b-1*, and *Mir92a-1*, is required to promote proliferation of orofacial development (Ning et al., 2013). Thus, these data confirm and validate the deep sequencing data. Other groups have performed miRNA expression profiling of the developing mouse orofacial region using microarrays (Mukhopadhyay et al., 2010) and have found similar differential expression across time for miRNAs that included *Mir133a* and *Mir133b*. Interestingly, *Mir23b* was not identified by microarray analysis. This may be due to the retrospective nature of microarray studies, as they are limited by the total number of miRNAs that are present on the microarray chip. This approach may exclude all of the transcribed miRNAs in the genome. By definition, the inclusion of miRNAs on microarray chips means that the miRNA has already been identified and annotated. Furthermore, because microarrays are generally based on genome sequence, they may not assay miRNAs that originate by post-transcriptional editing (de Hoon et al., 2010). In contrast, miRNA-seq is prospective: it identifies all miRNAs present in a sample, regardless of whether they have been annotated or not or whether they have experienced editing. Thus, miRNA-seq has the power to identify novel and edited miRNAs in addition to known miRNAs.

miRNA-seq was recently used on avian embryos, in which dissection of the chick, quail and duck FNPs followed by miRNA-seq identified 170 miRNAs that were differentially expressed between the three species (Powder et al., 2012). Interestingly, several miRNAs were found to be avian-specific, suggesting that miRNAs may have promoted the evolutionary diversification of facial shape and beak formation.

### Potential *Mir23b* and *Mir133b* functions and targets

Here we have shown that *Mir23b* is expressed in the developing face of mouse embryos and in the head of zebrafish embryos and that its overexpression in zebrafish embryos results in ectopic cartilage structures in the viscerocranium. This may indicate roles for *Mir23b* in regulating either patterning of the NCC-derived mesenchyme or later chondrogenesis. One of the interesting aspects our data analysis (Figure 1) is that *Mir27b* expression is also present in the developing midface, with its expression mirroring that of *Mir23b*. In mouse, *Mir23b* is part of a miRNA cluster that includes *Mir23b, Mir27b, Mir3074.1*, and *Mir24.1*. In zebrafish, this corresponds to *mir23b, mir27d*, and *mir24.1*. *mir23b* and *mir27b* are separated by less than 200 bp, though it is not clear that their expression is co-regulated. In the fetal mouse liver, the *Mir23b* cluster regulates cell fate switch between hepatocytes and bile duct cells by regulating expression of *Smad3, 4*, and *5* and thereby repressing TGF-β signaling (Rogler et al., 2009). This negative regulation of TGF-β signaling is interesting, as proper regulation of TGF-β signaling is crucial for proper craniofacial development (Behnan et al., 2005). Marfan syndrome results from aberrant TGF-β signaling due to the failure of fibrullin-1 to bind to and sequester the latent form of TGF-β (Neptune et al., 2003; Chaudhry et al., 2007). Conversely, loss of TGF-β signaling is also detrimental to midfacial development, with loss of numerous TGF family members leading to facial birth defects (Iwata et al., 2011), illustrating the importance of miRNA regulation. While our miRNA-seq data suggests that the two miRNAs are co-expressed, further analysis of embryonic expression and loss-of-function phenotypes for individual miRNAs and the clusters will be required to fully understand their function. This is especially true for *Mir23b* and *Mir27b*, as while both work concurrently to drive cardiomyocyte development from ES cells *in vitro, Mir23b* subsequently controls the later beating phenotype of differentiated cells while *Mir27b* functions to inhibit this event (Chinchilla et al., 2011; Wang et al., 2012). Our analysis of expression and function in zebrafish suggest that *mir23b* is expressed in the pharyngeal arch mesenchyme and potentially functions to promote proliferation of chondrocytes, such that when overexpressed, ectopic cartilage arises.

*Mir133b* resides in a cluster that includes *Mir206* and *Mir133b*. This family is referred to as myomiRs because they regulate genes involved in adult muscle formation. In addition, *Mir133b* is down-regulated in several cancers, including muscle rhabdomyosarcoma, osteosarcoma, and prostate, colorectal and gastric cancers (Namløs et al., 2012; Qin et al., 2012; Mo et al., 2013). In these abnormal contexts, *MIR133b* in human cervical carcinoma targets EGFR and FGFR1, similarly acting as a tumor suppressor (Namløs et al., 2012). Interestingly, both EGF and FGF signaling have been implicated in craniofacial development. Mutations in EGF signaling have been implicated in human clefting (Falagan-Lotsch et al., 2015) and cranial suture formation in mice (Rawlins et al., 2008). More roles have been ascribed for FGF signaling in craniofacial development, with FGFR1 specifically linked to skeletal dysplasias and craniosynostosis in both humans and mice, suggesting a mechanism by which *Mir133b* may regulate craniofacial development (Moosa and Wollnik, 2016). Changes in facial muscle can affect development of facial bone and cartilages (Reider et al., 2012). From our expression analysis, it is plausible that overexpression of *Mir133b* may lead to changes in facial muscle which affects subsequent viscerocranium development.

In addition, *Mir133b* is involved in the differentiation of dopaminergic neurons. Zebrafish *mir133b* is expressed in the midbrain at low levels and regulates *pitx3* to control dopaminergic neuron differentiation (Sanchez-Simon et al., 2010). In contrast, mice in which *Mir133b* has been inactivated have normal dopaminergic neuron numbers and normal PITX3 protein levels (Heyer et al., 2012) even though *Mir133b* can target the *Pitx3* message. This suggests the possibility that other *Mir133* family members with the same seed sequence (GGACCAAA; i.e., *Mir133a, Mir133c*) can compensate for loss of *Mir133b*. *Mir133b* is clustered in the genome with *Mir 206.1*, which does not have the same seed sequence but is predicted to bind some of the same targets in mouse including Histone Deacetylase 4, DNA Polymerase α, and Connexin43 (Anderson et al., 2006; Chen et al., 2006; Kim et al., 2006; Goljanek-Whysall et al., 2012). *mir133b* has the potential to target the same set of myogenic targets in zebrafish, but only Histone Deacetylase 4 contains a seed sequence for *mir133b*. More work is needed to distinguish between these possibilities. While we have not determined the numbers of dopaminergic neurons in zebrafish in which *mir133b* is over-expressed, we do see specific defects in cartilage differentiation, including hypoplasia of the ethmoid plate and specific gaps or missing cartilage in the viscerocranium, suggesting that *mir133b* may act non-cell autonomously to regulate cartilage differentiation. While there is little known about a direct role of *mir133b* on the development of the craniofacial cartilage, these results suggest that it is involved in both muscle and neuronal development, both of which can influence, either directly or indirectly, formation of the craniofacial complex. In summary, our results illustrate multiple MiRNAs are expressed in the developing face. This analysis will allow for more focused analysis of miR function in both zebrafish and mouse craniofacial development.

## Author contributions

Experiments were designed by DC and KA in consultation with JP. All mouse and zebrafish experiments were performed by HD with help from BE. The RNA-seq was performed by BE and JP with bioinformatics performed by PB and JH. HD, KA, and DC analyzed and interpreted the data, and wrote the manuscript. All authors commented on the manuscript.

## Funding

This work was supported by a grant from NIH/NIDCR (DE020076) to DC, KA, and JP.

### Conflict of interest statement

The authors declare that the research was conducted in the absence of any commercial or financial relationships that could be construed as a potential conflict of interest.
