# Supplementary material for: MicroRNA Profiling during Craniofacial Development: Potential Roles for Mir23b and Mir133b
Source: Front Physiol. 2016 Jul 14;7:281. doi: 10.3389/fphys.2016.00281 (PMC4943961; doi:10.3389/fphys.2016.00281)
Supplement: Supplementary file 1 [file DataSheet1.PDF]

MOUSE

Mir15a-down	GCTATCATAGGAGCTATG
Mir15a-up	CTATTGAGGTGCTAGGAG
Mir20a-down	CTAACCATAGACCAGTGCTC
Mir-20a-up	GATGGTGGCCTGCTATTTAC
Mir23b-down	CTGGTGAGCATCTTCGAAG
Mir23b-up	GAGGACACTCAGCACATG
Mir24.1-down	CTCAGGCACTTACAGATG
Mir24.1-up	GTCGCTGGCAAGATGATG
Mir27b-down	AGTGACCACCAGGCAGTG
Mir27b-up	CCTTGTGGCTCTTTGGAA
Mir128.2-down	CAGCATGACAATTAGCAC
Mir128.2-up	CTCCTGACTATGCAGCGTG
Mir130a-down	CTGACTGGTGCTCAGGTG
Mir130a-up	GTCTCACTTGGCTCTGCA
Mir130b-down	CTATGATCCAAGCACTGAC
Mir130b-up	CATGCGTTCTAGGTCTAAG
Mir133b-down	GAGCATGTGACCTGTGAAC
Mir133b-up	GTGACAGGCTTGGACAAGTG
Mir206-down	TGTAGCCAAGGAACGAAG
Mir206-up	TTCTGCGTGACAAGTGCCT
Mir335-down	CACTGAATATCAAGTCTGG
Mir335-up	GATTGACTTGATGACTGC
Mir411-down	GATGGTATGGACTGGATGGT

Mir411-up	CTTCTTGGACCTGCCCTCAGG
Mir666-down	GTACGTCAGCAGCATGGAAC
Mir666-up	GTCACGGATCCAGAGCTG

ZEBRAFISH

mir15a.1-down	ACACGTAGTCTTGCTGCTG
mir15a.1-up	CGAGAACCGTGCGTGTGT
mir15a.2-down	CTTGCTTCAGCAGCACGA
mir15a.2-up	GGAGTCACTGGCTCTGTG
mir15b-down	GGATTTACGACACAAGCA
mir15b-up	CCAATCTGATCCTAGAGA
mir27b-down	CTTCACATCTGTAGCCAT
mir27b-up	TGAAGGTAGAAGAAGAGC
mir30c-down	GCTCTGTTACAGTGAACA
mir30c-up	CGTGATATTCAGGTATGT
mir130a-down	ACGCTGATGACCCTACCT
mir130a-up	GAGCACCCTGATTGTGTA
mir301a-down	CCAGCAATAGGAGACGAC
mir301a-up	AGGTAGGGTCATCAGCGT
