# Supplementary material for: MicroRNA Profiling during Craniofacial Development: Potential Roles for Mir23b and Mir133b
Source: Front Physiol. 2016 Jul 14;7:281. doi: 10.3389/fphys.2016.00281 (PMC4943961; doi:10.3389/fphys.2016.00281)
Supplement: Supplementary file 2 [file Image1.pdf]

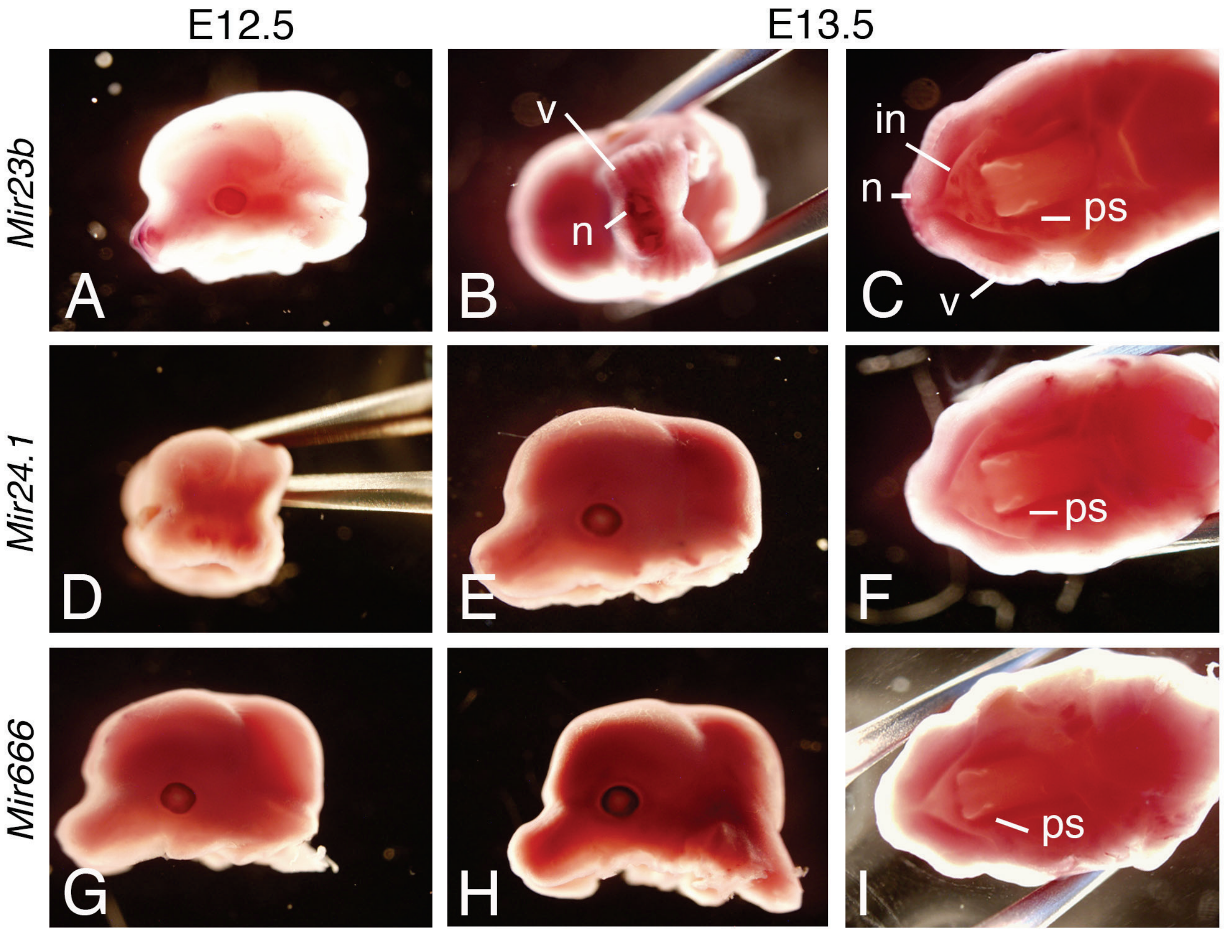

Supplemental Figure 1. Whole mount ISH analysis of *Mir23b, Mir24-1 and Mir666* expression in mouse facial structures at E12.5 examined using LNA riboprobes. A-C. *Mir23b* is expressed in head epithelium and mystacial vibrissae (v)(A), nasal epithelium (ne)(A,B) and edges of the palatal shelves (ps) (C). **D-F.** *Mir24-1* is expressed along the edges of the palatal shelves (F). **G-I.** This expression pattern is shared with *Mir666* (I). in, incisor.

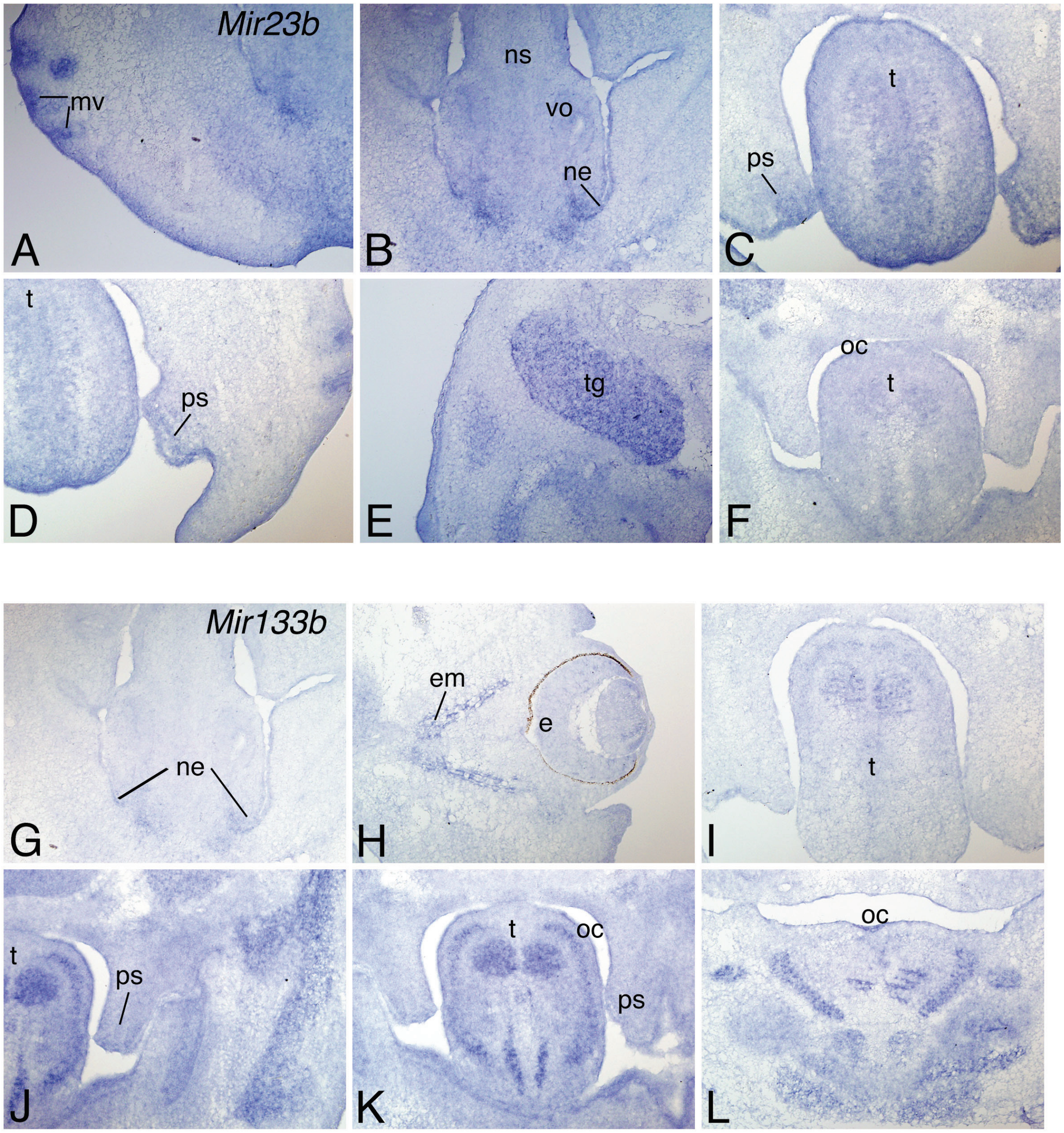

Supplemental Figure 2. Sectional ISH analysis of *Mir23b* and *Mir133b* expression in mouse facial structures at E12.5 examined using LNA riboprobes. Frozen frontal sections are shown. **A-F.** *Mir23b* is expressed in head epithelium and mystacial vibrissae (mv)(A), nasal epithelium (ne)(B), palatal shelves (ps) and tongue (t) epithelium (C, D) and in the trigeminal ganglion (tg). **G-L.** *Mir133b* is weakly expressed in the nasal epithelium (ne)(G), and more highly expressed in the overall head musculature, including the extrinsic eye muscles (em)(H) and in the tongue musculature (I-L). oc, oral cavity.

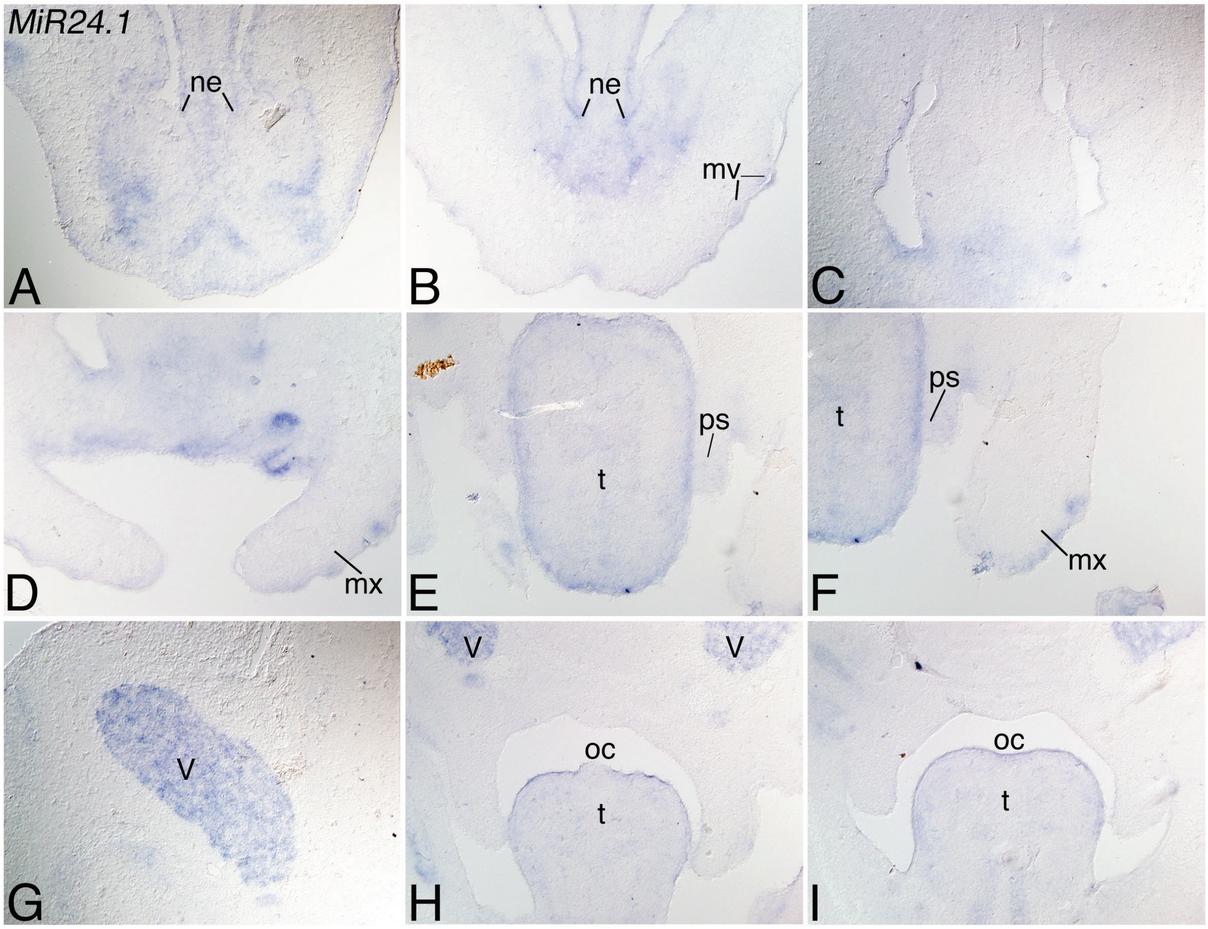

Supplemental Figure 3. Sectional ISH analysis of *MiR24.1* expression in mouse facial structures at E12.5 using a pri-miRNA probe. Frozen frontal sections are shown. *MiR24.1* is weakly expressed in nasal epithelium (ne; A, B), mystacial vibrissae (mv; B), trigeminal ganglion (V; G, H), and tongue (t; E, F, H, I) but not in palatal shelves (ps; E, F). mx, maxilla; oc, oral cavity.

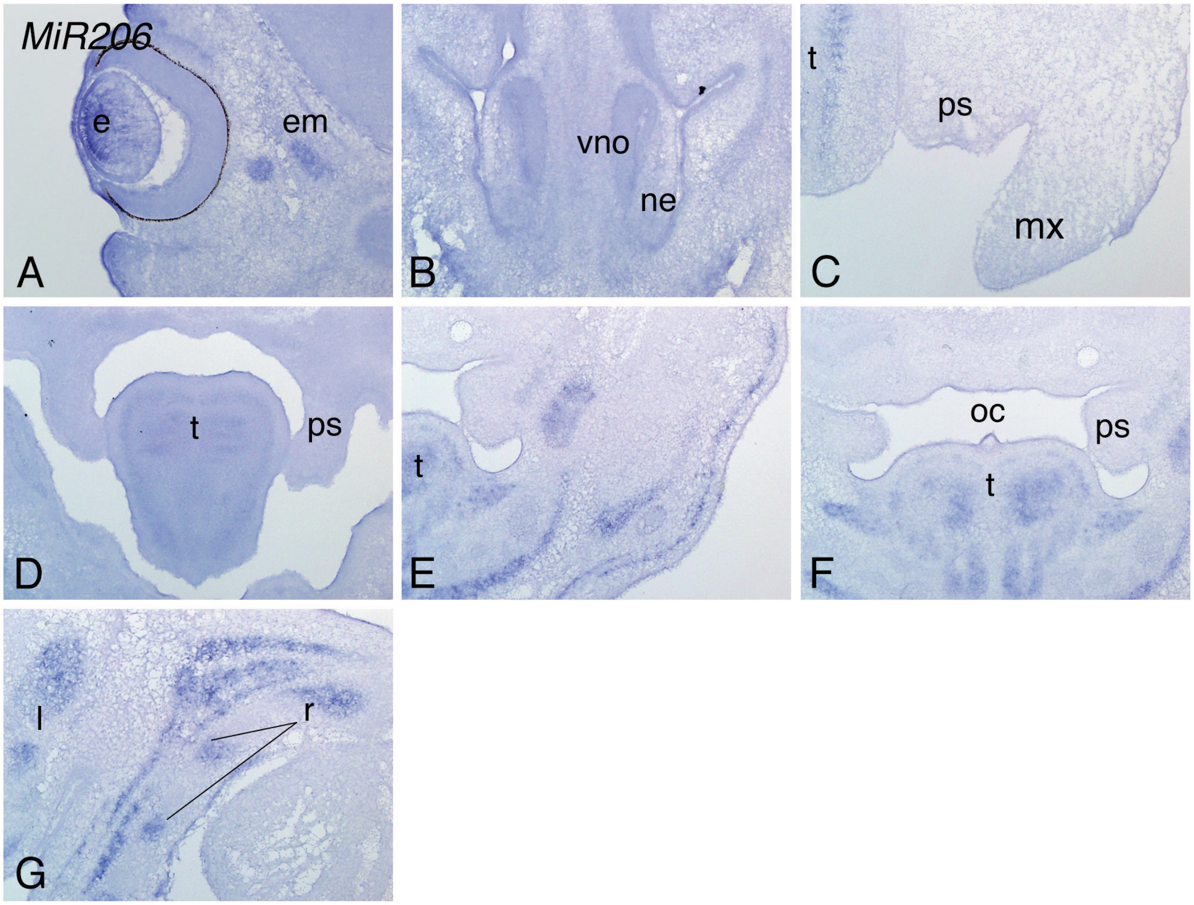

Supplemental Figure 4. Sectional ISH analysis of *MiR206* expression in mouse facial structures at E12.5 using a pri-miRNA probe. Frozen frontal sections are shown. *MiR206* is expressed in the lens and cornea of the eye (e, A) and facial muscles, including those associated with the tongue (t, E, F), eye (em, A) and jaw (E, F). Expression is also observed in the early cartilage condensations of the ribs (r) and limb (l)(G). mx, maxilla; nasal epithelium; ps, palatal shelves; oc, oral cavity; vno, vomeronasal organ.

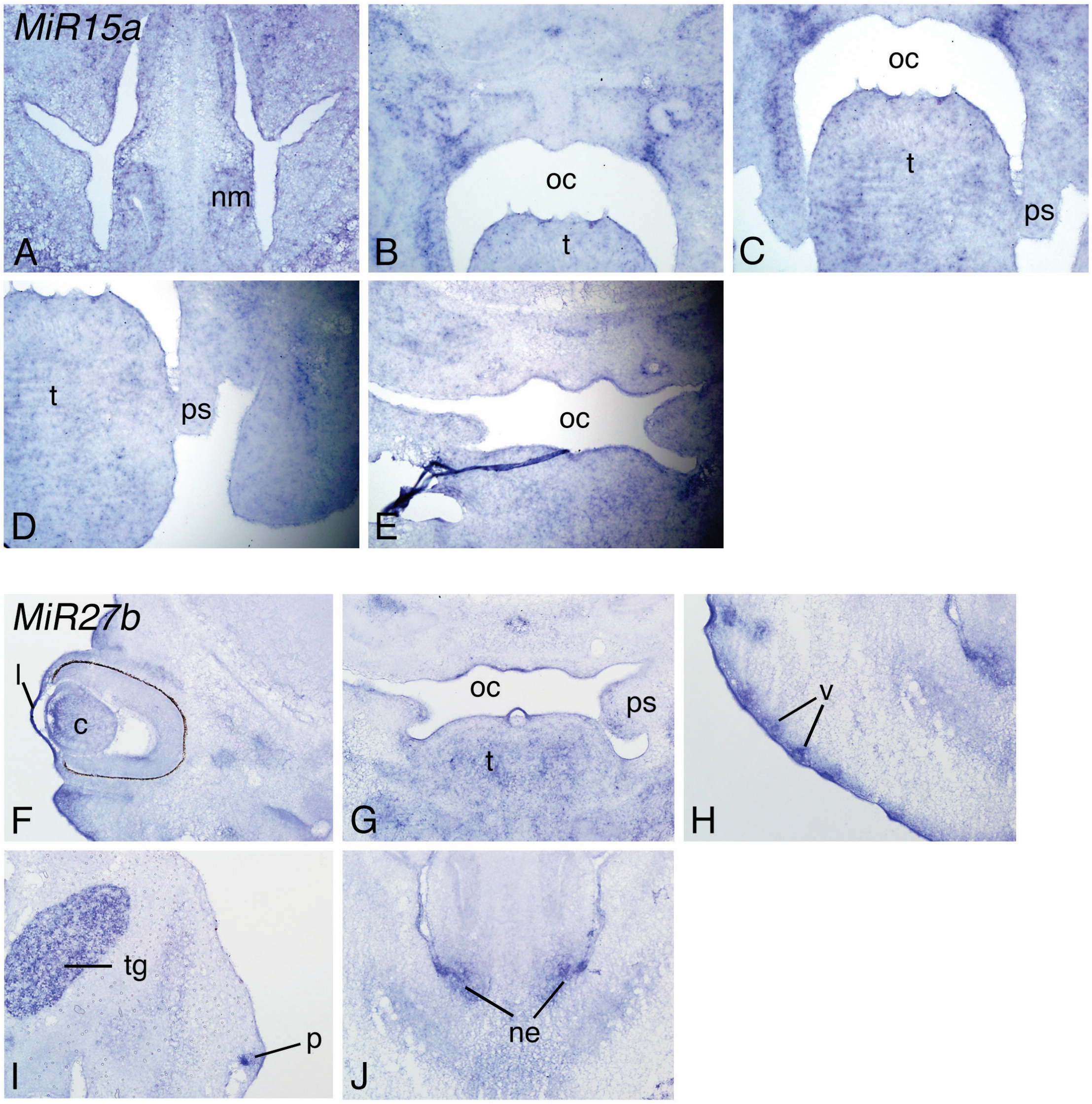

Supplemental Figure 5. Sectional ISH analysis of *MiR15a* and *MiR27b* expression in mouse facial structures at E12.5 using pri-miRNA probes. Frozen frontal sections are shown. **A-E.** *MiR15a* is expressed in the vomeronasal organ (vo) and nasal mesenchyme (nm)(A), palatal mesenchyme (B) and tongue (t) musculature (C-E). Epithelia in the oral cavity (oc) and tongue also shows expression (B-E). **F-J.** *MiR27b* is expressed in the lens (l) and cornea (c) of the eye (F), epithelia of both the oral cavity (oc) and the palatal shelves (ps)(G), head epithelium and vibrissae (v) (H), the trigeminal ganglion (tg) and ear pinna (p)(I) and the nasal epithelium (ne)(J).

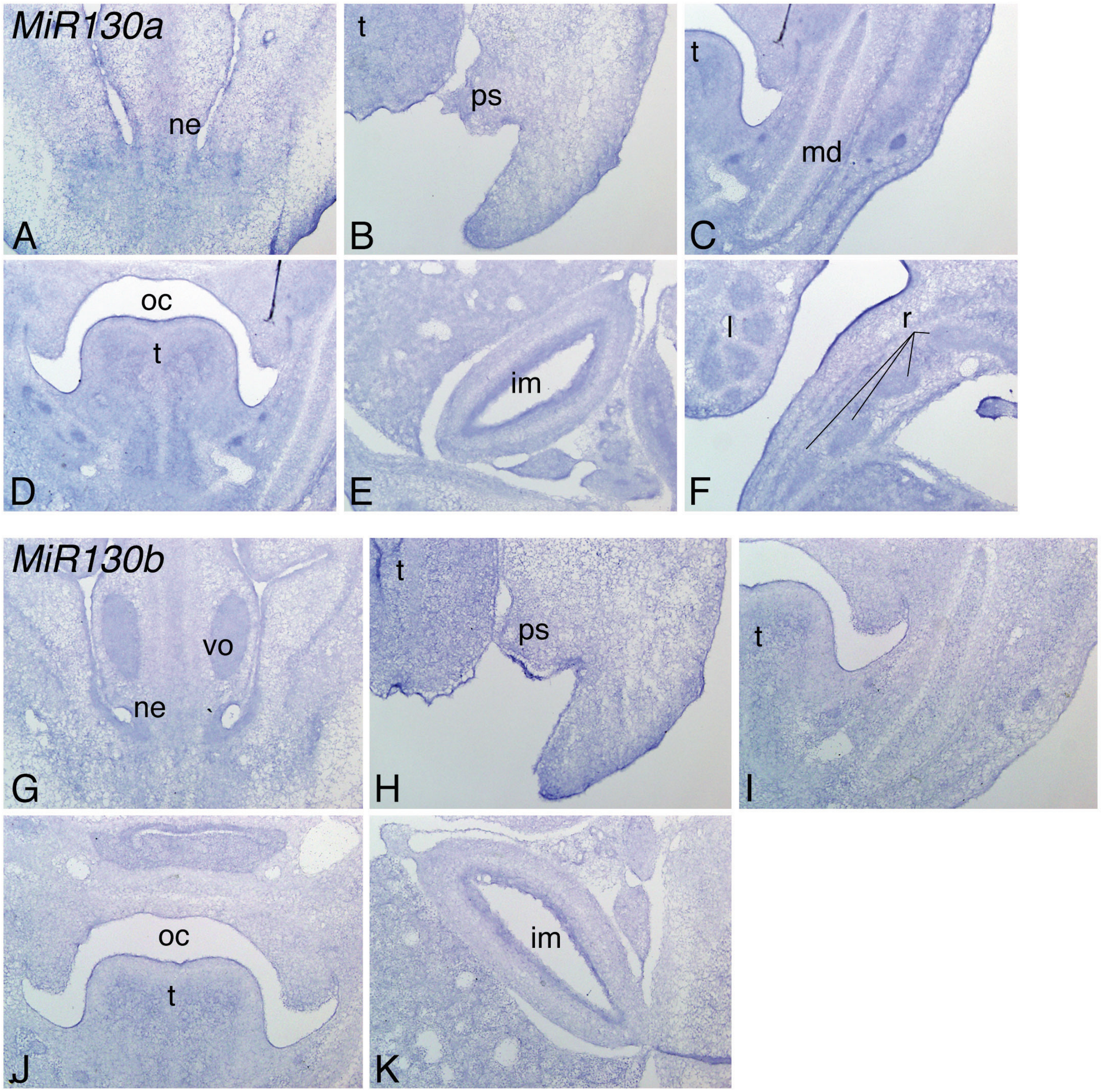

Supplemental Figure 6. Sectional ISH analysis of *MiR130a* and *MiR130b* expression in mouse facial structures at E12.5 using pri-miRNA probes. Frozen frontal sections are shown. **A-F.** *MiR130a* is weakly and broadly expressed in the nasal epithelium (A), in nerve bundles in the lower jaw (D) and in the bone of the mandible (md) but not in the surrounding periosteum (C). Weak expression is also observed in the intestinal mucosa (im)(E). **G-K.** *MiR130b* is expressed in the anterior epithelium of the palatal shelves (ps)(H) and weakly in the tongue musculature (t)(J) and intestinal mucosa (im). ne, nasal epithelium; oc, oral cavity; vo, vomeronasal organ.

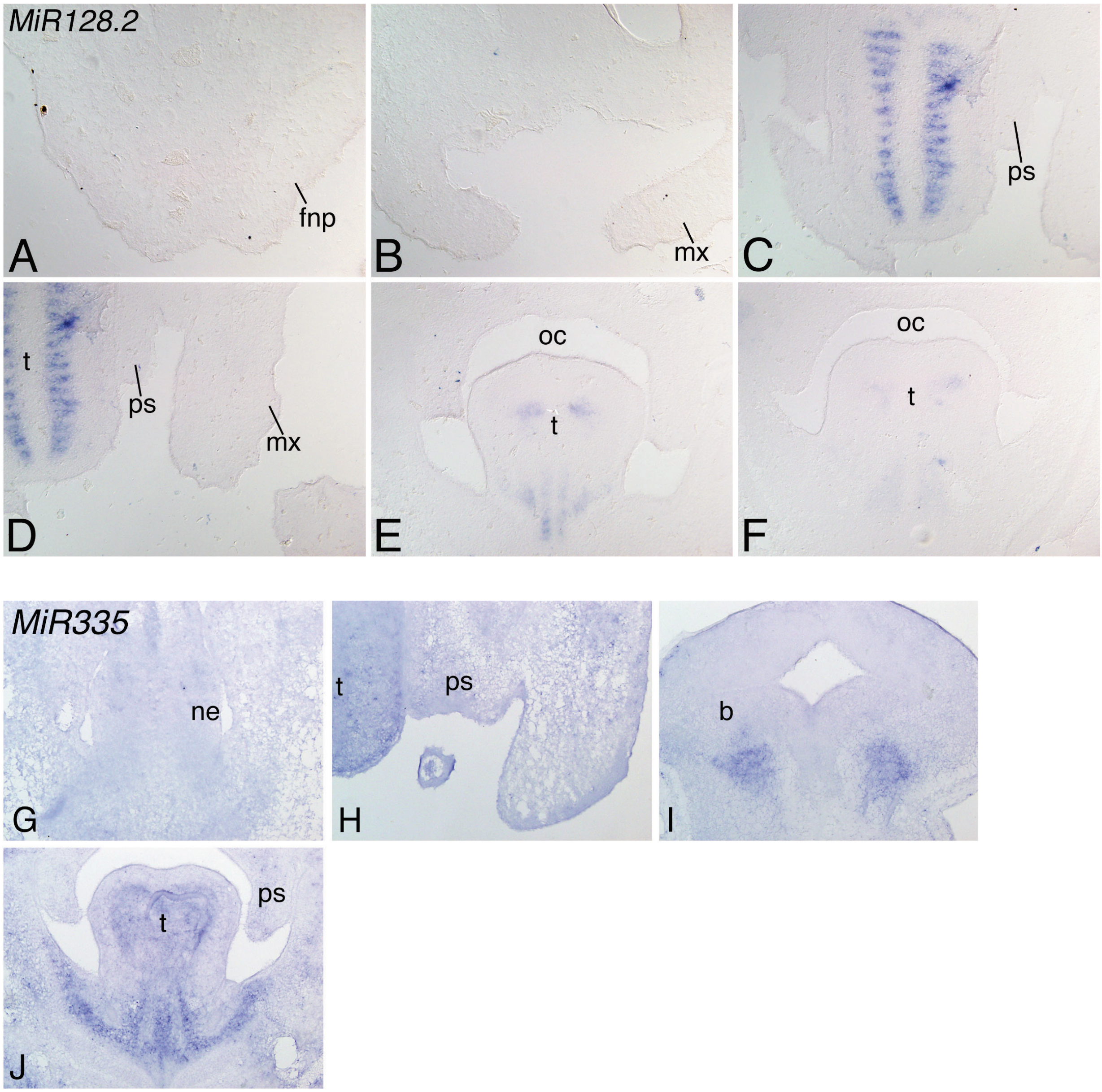

Supplemental Figure 7. Sectional ISH analysis of *MiR128.2* and *MiR335* expression in mouse facial structures at E12.5 using pri-miRNA probes. Frozen frontal sections are shown. **A-F.** *MiR128.2* is expressed in the tongue musculature (t) (C, D, E). **G, J.** *MiR335* is expressed in the brain (J) and tongue musculature (t)(K). oc, oral cavity; fnp, frontonasal prominence; mx maxilla; ps, palatal shelves.

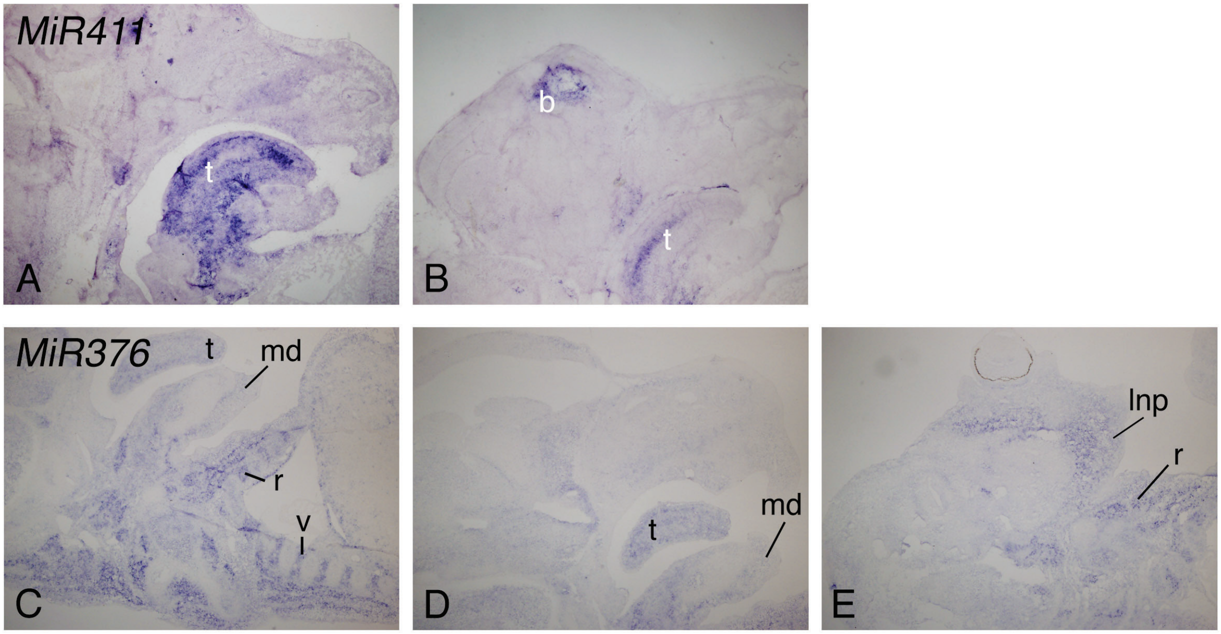

Supplemental Figure 8. Sectional ISH analysis of *MiR411 and MiR376* expression in mouse facial structures at E12.5 using pri-miRNA probes. Frozen frontal sections are shown. **A, B.** *MiR411* is strongly expressed in the tongue mesenchyme (t; A, B) and in a region of the midbrain (b; B). **C-E.** *MiR376* is expressed in the mesenchyme of the tongue (C, D), mandible (md; C), ribs (r) and vertebrae (v) (C) and mesenchyme of the lateral nasal prominence (lnp, E).

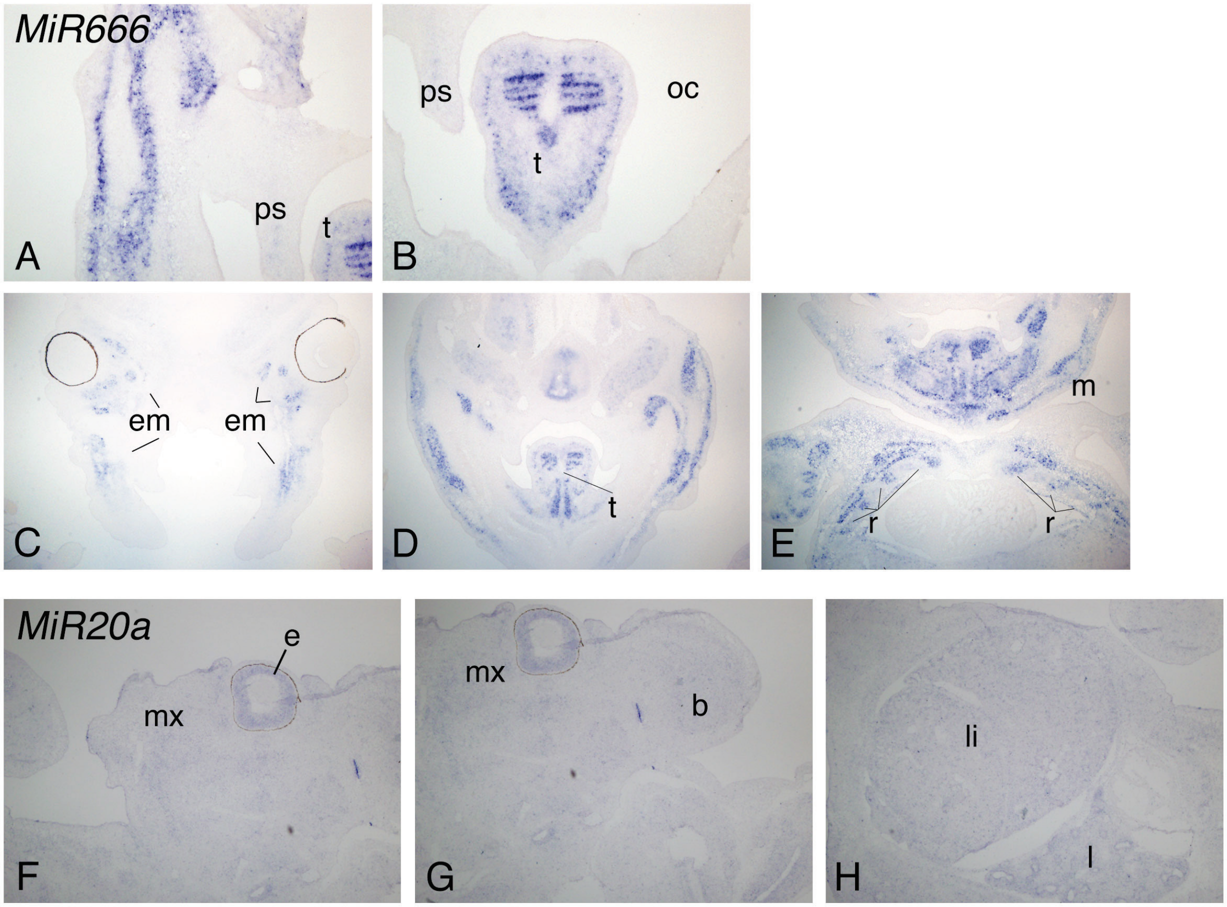

Supplemental Figure 9. Sectional ISH analysis of *MiR666 and MiR20a* expression in mouse facial structures at E12.5 using pri-miRNA probes. Frozen frontal sections are shown. **A-E.** *MiR666* is expressed in trunk muscle (B, F), tongue (t) muscle (C, E, F), extrinsic eye muscles (em)(D), facial muscles (m)(E) and in the developing ribs (r) (F). **F-H.** *MiR20a* is very weekly expressed in the eye (G, H) and in lung epithelia (l) and liver (li). Expression in other craniofacial structures is not observed.

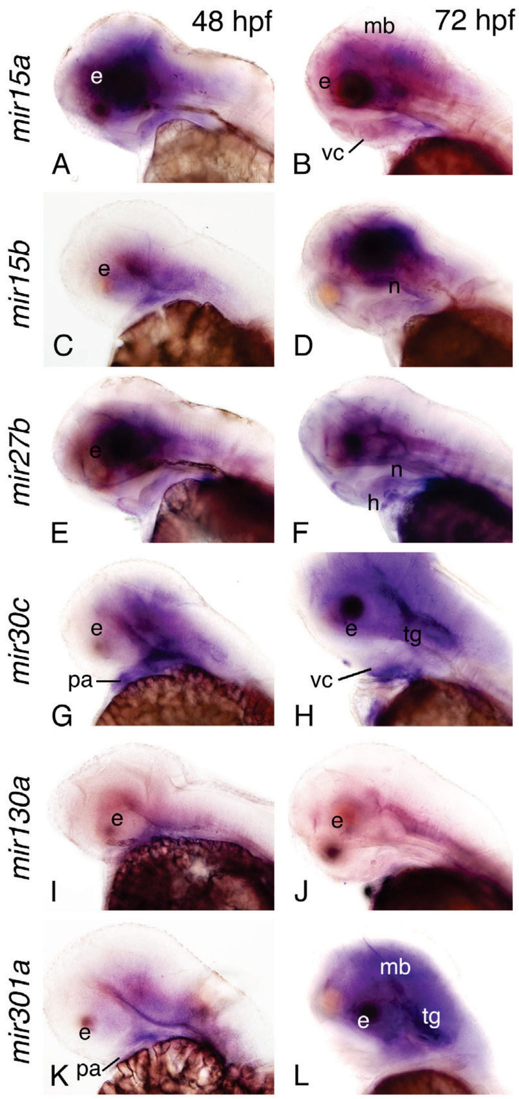

Supplemental Figure 10: Whole mount ISH analysis miRNA expression in zebrafish embryos at 48 and 72 hpf. ISH analysis was performed using LNA probes. **A, B.** *mir15a* is expressed in the viscerocranium (vc) as well as the midbrain (mb) at 48 and 72 hpf. **C, D.** *mir15b* is expressed within the forming pharyngeal arch region at 48 hpf and expands to the neurocranium (n) and midbrain at 72 hpf. **E, F.** *mir27b* is expressed within the neurocranium, brain and heart (h) region at 48 and 72 hpf. **G, H.** *mir30c* expression is observed in the pharyngeal arches (pa) and brain at 48 hpf, and in the viscerocranium and trigeminal primordial (tg) at 72 hpf. **I, J.** *mir130a* is weakly expressed in the pharyngeal arch region at 48 hpf, and in the brain at 72 hpf. **K, L.** *mir301a* is expressed in the pharyngeal arches at 48 hpf and expands to the midbrain and trigeminal ganglia at 72 hpf. e, eye.
